# Seroprevalence of tick-borne infections in blood donors in Europe: a systematic review

**DOI:** 10.1016/j.nmni.2025.101597

**Published:** 2025-05-10

**Authors:** Sophie Mathys, Nejla Gültekin, Zeno Stanga, Ismail Ülgür, Patricia Schlagenhauf

**Affiliations:** a University of Zürich, Epidemiology, Biostatistics and Prevention Institute, Zurich, Switzerland; b Centre of Competence for Military and Disaster Medicine, Swiss Armed Forces, Bern, Switzerland; c University of Zürich, Epidemiology, Biostatistics and Prevention Institute, WHO Collaborating Center for Travellers' Health, Competence Centre for Military Medicine Biology, Zurich, Switzerland

**Keywords:** Tick, Infection, Seroprevalence, Europe, Blood Donor, Transfusion

## Abstract

**Background:**

Tick-borne infections (TBIs) pose an increasing threat to public health and recent research shows a wide range of infections transmitted to humans by tick bite. This situation may have an impact on blood safety in the context of transfusion-transmitted TBIs. We aimed to assess the seroprevalence of TBIs in blood donors in Europe in the period 2000 to 2024.

**Methods:**

This systematic review followed PRISMA guidelines. We searched PubMed, Embase, MEDLINE, Scopus, CINAHL, and national reporting systems up to April 2024 using keywords related to TBIs, Europe and epidemiology. Two reviewers independently screened and selected studies, focusing on seroprevalence of TBIs in European blood donors from 2000 to 2024. Data extraction and risk of bias assessment were performed.

**Results:**

The search yielded 5304 articles, of which 56 met the inclusion criteria. We added one article after citation search. The included studies encompassed 19 European countries and 11 different TBIs. The most studied pathogen was *Borrelia* spp. The majority of studies used antibody detection as a diagnostic technique. The highest seroprevalence rates were observed for *Tick-Borne Encephalitis Virus* (*TBEV*), *Bartonella* spp., *Rickettsia* spp. and *Borrelia* spp. with pathogen seropositivity rates, in some studies, of over 20 % depending on the pathogen and the vaccination status of included individuals.

**Conclusions:**

This study highlights the need to focus on a wider range of tick-borne pathogens to better understand the epidemiological landscape of TBIs. Additionally, incorporating Nucleic Acid Amplification Testing of donated blood will improve the ability to differentiate between past exposure and potential infectivity, to allow for an improved assessment of TBI transmission risk in transfusion medicine.

## Highlights

• Serological and molecular evidence of 11 tick-borne infections has been detected in blood donors' sera in Europe.
• Several tick-borne pathogens demonstrated significant seroprevalences of over 20 %.
• The majority of studies used antibody detection as a diagnostic technique.
• Need for a broader research focus on a wider range of tick-borne pathogens.
• Incorporating Nucleic Acid Amplification Testing allows for the detection of pathogen presence with potential infectivity.

## 1 Introduction

Tick-borne infections (TBIs) represent a significant and growing health concern across Europe. Recently, European tick surveillance has focused on the main risks associated with *Ixodes ricinus,* which transmits Lyme Borreliosis (LB) and Tick-Borne Encephalitis (TBE), and with *Hyalomma marginatum*, which is responsible for transmitting Congo Crimean Hemorrhagic Fever (CCHF) [1]. An increase in TBIs has been reported in various European countries, including the spread of TBE within the European Union and parts of Russia [2,3] and the rise of LB in the Netherlands [4] and across Europe [5]. Climate change is often suggested as an important driver of increased TBIs [6]: Changing climatic conditions, such as rising temperatures and humidity levels, tend to increase habitat suitability for specific tick species in Europe [7] and contribute to the expansion of their habitats [3]. Factors such as human behavior, movement, land use and reforestation are also likely to contribute to the increased rates of arboviral transmission [8]. Furthermore, imported cases of TBIs due to international travel to Europe have been reported [[9], [10], [11]]. Changes in case reporting and definitions have also led to the increase in the United States [12], and it is plausible that similar factors are contributing in Europe.

The heterogeneity of TBIs encompasses a wide range of pathogens, such as bacteria (including *Borrelia* spp., *Rickettsia* spp. and *Francisella tularensis)*, viruses (including *Tick-Borne Encephalitis Virus* (*TBEV*) and *Crimean-Congo Hemorrhagic Fever Virus* (*CCHFV*)) and parasites (including *Babesia* spp.) [13,14]. Traditionally, reviews and studies on TBIs have often been narrow in scope, focusing on specific diseases such as Lyme disease or TBE or have been confined to specific geographic areas, such as individual countries. However, the landscape of TBIs is evolving, with emerging and re-emerging infections being identified across a broader geographical range, such as the increasing occurrence of Babesiosis in southeastern, central, and northeastern Europe [15], CCHF in southeastern Europe [16,17] or TBE in the European Union/European Economic Area [2]. Even though these infections often present with non-specific clinical symptoms, they can still pose a significant threat to health, with mortality rates varying depending on the specific pathogen involved. For instance, while a diagnosis of Lyme neuroborreliosis seems to have no effect on long term survival or health [18], the mortality rate for TBE varies between 0.5 % and 35 %, depending on the subtype [19], and reaches 30 % in CCHF [20]. Co-infections of TBIs (such as of *Spotted Fever Group Rickettsiae* and *Anaplasma phagocytophilum*) have been shown to be underdiagnosed and are associated with increased morbidity in patients, presenting a significant health risk [21]. This shift necessitates a broader and more comprehensive approach to understanding the epidemiology of these diseases. Furthermore, many studies on the seroprevalence of TBIs have so far primarily focused on high-risk groups, such as forestry workers, farmers, and veterinarians.

Blood donors provide a valuable population for measuring seroprevalence in the general population. However, the ‘Healthy Donor Effect’ may limit the representativeness of this group, as individuals who donate blood tend to be healthier compared to the broader population [22]. Despite this, blood donors are still a practical choice for seroprevalence studies due to their accessibility, the wide geographical coverage of donation centers, and the availability of demographic data, which provide a useful framework for allowing estimates of exposure to pathogens and the prevalence of infections, including asymptomatic or subclinical cases. The detection of TBIs in donated blood also raises concerns about the potential risk of transfusion-transmitted TBIs (TTTBIs), which have been reported most frequently in North America [23]. *Babesia* spp. represent the greatest concern [24] – however, other tick-borne pathogens, such as *Borrelia burgdorferi,* could also cause transfusion-transmitted infections, although knowledge about this is still limited [25]. Up to this date, only one autochthonous case of transfusion-transmitted *Babesia* spp. infection has been reported in Europe [26]. In a study conducted in Finland, covering the period from 1959 to 1987, two cases of transfusion-transmitted TBE were documented. These cases occurred as a result of blood transfusions from donors who were in the viremic phase of a *TBEV* infection. However, further details regarding these cases, such as the clinical outcomes of the patients, the specific circumstances of the transfusions, and any follow-up measures taken, are not available in the published literature [27]. Despite increased awareness of potential TTTBI cases, interventions to protect blood supply have yet to be expanded.

Despite growing public attention on the topic, significant gaps remain in our understanding of exposure to tick-borne pathogens in the European population and the risk of TTTBIs. This study aims to provide an overview and an approach to filling this knowledge gap by systematically reviewing the seroprevalences of TBIs in European blood donors. The findings will not only contribute to the current knowledge in the field but may also have practical implications for public health measures.

## 2 Methods

### 2.1 Data sources and search strategy

This systematic review was conducted following standard methods and reported according to the PRISMA 2020 statement. The review was registered with PROSPERO (CRD42024513422).

Articles were retrieved from the following databases: PubMed, Embase, MEDLINE, Scopus and CINAHL on May 1, 2024.

We selected the keywords based on the population, geographical regions, pathogens, and epidemiological outcomes relevant to the research question. The search strategy for the databases is provided in (Supplementary File 1). Additionally, we obtained publicly available surveillance data on the incidence of TBIs to develop country-level estimates from the following websites: ECDC, government public health agencies and institutes in Europe with TBI surveillance programs, and annual reports (Supplementary File 2).

Studies in English, German, French, Spanish, Italian, and Finnish were searched. Europe was defined to include 50 countries and autonomous regions as per the UN definition [28].

### 2.2 Study selection, inclusion and exclusion criteria and screening of articles

For this review, we considered any study including case reports, cohort studies, and reviews. Citations were imported and duplicates excluded using the reference manager Zotero. Title and abstract screening were performed using the reference manager Rayyan to identify potentially relevant publications.

Eligible articles were further subjected to full-text screening by two reviewers (SM and PS). Disagreements regarding study inclusion were resolved through discussion among the review authors. Citation screening was conducted to identify additional potentially relevant publications.

Inclusion criteria included studies reporting seroprevalence of any TBI in human blood donors in Europe. We included studies conducted between January 1, 2000, and April 30, 2024. Exclusion criteria included animal studies, studies on alimentary transmission of infections, studies concerning non-European countries, and studies reporting data from before 2000.

Given the large volume and heterogeneity of data from our initial literature search, we narrowed the focus of our research question to investigate seroprevalence of TBIs in blood donors only, a study group representing a healthy, averagely exposed population.

### 2.3 Data extraction and analysis

The primary outcome of this study was seroprevalence of TBIs in blood donors. A data extraction template was developed using the online data extraction software SRDR+ and then exported to an Excel worksheet. The following data were extracted from the included articles: authors, study design, sample characteristics, year of study, country, years of data collection, pathogens, seroprevalence and diagnostic methods. We conducted a descriptive synthesis of the data. Due to the heterogeneity of the data, a meta-analysis was not possible.

### 2.4 Risk of bias assessment

The JBI Critical Appraisal Checklist for Studies Reporting Prevalence Data [29] was used to assess the risk of bias of this study. The JBI Checklist was chosen because it is specifically designed to assess risk of bias in prevalence studies. The included studies were appraised for inclusion, exclusion or need of further information.

### 2.5 Consultation with blood donation services

To gather more information on infectious disease screening, particularly TBIs, in donated blood products, we consulted recent reports from Swiss and European blood donation services. Additionally, a conversation was held with a representative from the Swiss Blood Donation Service.

## 3 Results

**Fig. 1:**
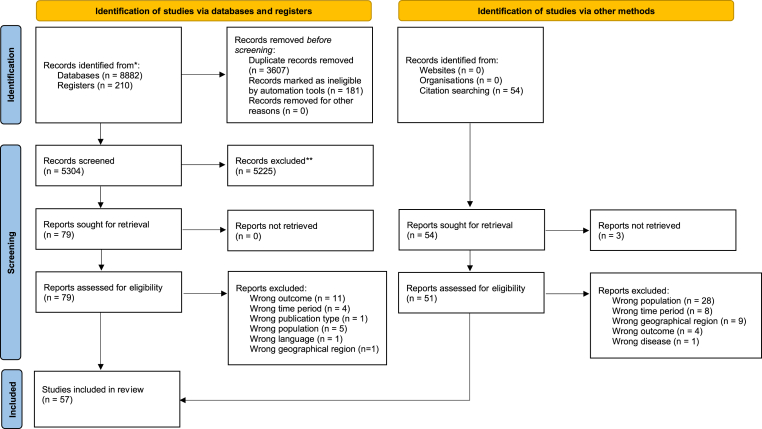
PRISMA Flowchart demonstrating the Study Selection Process of *Seroprevalence of Tick-Borne Infections in Blood Donors in Europe: a Systematic Review*.

The initial literature search yielded a total of 5304 articles. After eliminating duplicates and screening titles and abstracts, 79 articles were identified as relevant or potentially relevant for further screening. After full-text screening of these studies, 56 articles met the inclusion criteria. A citation search identified one additional relevant study. In total, 57 publications were included in this study for analysis and data extraction. The detailed flow of the systematic review study selection process is shown in Fig. 1.

### 3.1 Study and sample characteristics

**Fig. 2:**
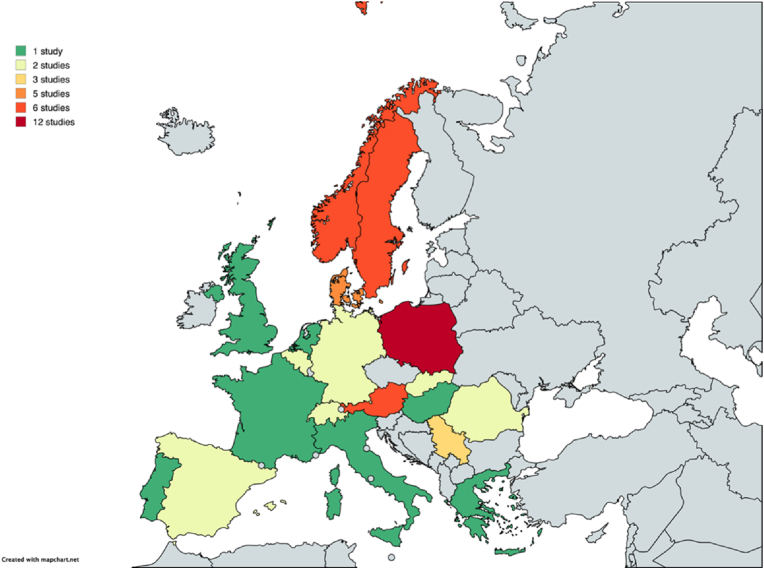
European Countries in which Prevalence Studies on Tick-borne Infections of Blood Donors have been conducted and Number of Studies included per Country. Author Note: Map lines delineate study areas and do not necessarily depict accepted national boundaries.

The studies were conducted between January 2000 and April 2024 in 19 European countries (Fig. 2). Most included studies were conducted in the following countries: Poland (12 studies); Norway and Sweden (6 studies each); Austria and Denmark (5 studies each).

Heterogeneity was observed in terms of population, study design, exposure, and pathogen. Overall, the studies encompassed 49′395 blood donors, with sample sizes per study from 30 to 9′328 participants.

In 28 of 57 studies, seroprevalence was studied exclusively in blood donors; in the remaining 29 studies, blood donors served as the control group against which a different population was examined. The selection criteria of the respective study populations varied between studies, as shown in (Table 1, Additional Information).

**Table 1:** Main Findings of Papers describing Seroprevalence of Tick-Borne Infections in Blood Donors in Europe.

Nr.	Authors	Title	Year of publication	Country	Population examined	Year(s) of data collection	Sample size	Examined pathogen(s)	Diagnostic technique(s)	Found seroprevalence(s)	TTI	Additional information	Reference
1	Ackermann-Gäumann et al.	Comparison of four commercial IgG-enzyme-linked immunosorbent assays for the detection of *Tick-Borne Encephalitis Virus* antibodies	2019	Switzerland	Blood donors only	2014–2015	876	*TBEV*	Antibody (IgG)	23.7–53.3 %	No	Seroprevalence rates depended on screening test. Population: healthy blood donors from endemic and non-endemic regions. Seroprevalence rates depended on vaccination status.	30
2	Ackermann-Gäumann et al.	Prevalence of anti-Tick-Borne Encephalitis *Virus* (*TBEV*) antibodies in Swiss blood donors in 2014–2015	2023	Switzerland	Blood donors only	2014–2015	9328	*TBEV*	Antibody (IgG)	Individuals with previous *TBEV* infection: 0.34 %, thereof 59.4 % seropositive; 81.1 % (vaccinated individuals); 5.6 % (non-vaccinated individuals)	No	Samples collected from 7 different blood transfusion services, regions selected to include endemic regions. Seroprevalence rates depended on vaccination status.	31
3	Albinsson et al.	Seroprevalence of *Tick-Borne Encephalitis Virus* and vaccination coverage of Tick-Borne Encephalitis, Sweden, 2018 to 2019	2024	Sweden	Blood donors only	2018–2019	2700	*TBEV*	Antibody (IgG)	27.50 %	No	Seroprevalence rates varied depending on region and vaccination status.	32
4	Banović et al.	Shared odds of *Borrelia* and *Rabies Virus* exposure in Serbia	2021	Serbia	Blood donors as control group	2019	30	*Borrelia afzelii*	Antibody (IgG)	6.67 %	No	Population: healthy blood donors, no profession associated with risk of exposure to rabies virus.	33
5	Banović et al.	*Tick-Borne Encephalitis Virus* seropositivity among tick infested individuals in Serbia	2021	Serbia	Blood donors as control group	2020	50	*TBEV*	Antibody (IgG)	4 %	No	Population: healthy blood donors, not exposed to tick bites, no previous vaccination against TBE.	34
6	Barreiro-Hurlé et al.	Seroprevalence of Lyme disease in southwest Asturias	2020	Spain	Blood donors as control group	2014	316	*Borrelia burgdorferi*	Antibody (IgG)	5.10 %	No		35
7	Bazovska et al.	Reported incidence of Lyme disease in Slovakia and antibodies to *B. burgdorferi* antigens detected in healthy population	2005	Slovakia	Blood donors only	n/a	250	*Borrelia burgdorferi*	Antibody (IgG)	4.4 %–15.6 % (cumulatively 12.8 %)	No	Three different screening tests were used.	36
8	Bloch et al.	Molecular Screening of Blood Donors for *Babesia* in Tyrol, Austria	2023	Austria	Blood donors only	2021	7972	*Babesia microti, B. divergens, B. duncani, B. venatorum*	PCR	0 %	No	Individuals excluded: direct-, autologous-, and apheresis platelet/plasma donors; individuals with a reported tick bite in the 4 weeks prior to donation.	37
9	Borawski et al.	Assessment of *Coxiella burnetii* presence after tick bite in north-eastern Poland	2020	Poland	Blood donors as control group	2015–2018	20	*Coxiella burnetii*	Antibody (IgG)	0 %	No	Population: honorary blood donors, no history of tick bite.	38
10	Borawski et al.	Prevalence of *Spotted Fever Group Rickettsia* in North-Eastern Poland	2019	Poland	Blood donors as control group	2015–2018	20	*Rickettsiae* spp*., Borrelia burgdorferi, Anaplasma phagocytophilum, TBEV*	*SFGR*: Antibody (IgG); *B. burgdorferi*: Antibody (IgG); *A. phagocytophilum*: PCR; *TBEV*: Antibody (IgG)	0 %	No	Population: honorary blood donors, no history of tick bite. No information on TBE vaccination status.	39
11	Brouqui et al.	Ectoparasitism and vector-borne diseases in 930 homeless people from Marseilles	2005	France	Blood donors as control group	2000–2003	467	*Rickettsia conorii, R. typhi, R. akari, R. felis*	Antibody (IgG)	*R. conorii*: 1 %; *R. typhi*: 0.2 %; *R. akari*: 0 %; *R. felis*: 0.6 %	No	Population: sex- and age-adjusted controls.	40
12	Busson et al.	Evaluation of commercial screening tests and blot assays for the diagnosis of Lyme borreliosis	2012	Belgium	Blood donors as control group	2007–2009	50	*Borrelia burgdorferi, B. afzelii, B. garinii*	Antibody (IgM and IgG)	2 %	No		41
13	Carlsson et al.	Subclinical Lyme borreliosis is common in south-eastern Sweden and may be distinguished from Lyme neuroborreliosis by sex, age and specific immune marker patterns	2018	Sweden	Blood donors only	2012	1113	*Borrelia afzelii, B. garinii*	Antibody (IgG)	8 % (individuals with no previous history of Lyme Borreliosis), 12 % (with previous Lyme Borreliosis); 1 % (undetermined)	No		42
14	Chmielewska-Badora et al.	Serological survey in persons occupationally exposed to tick-borne pathogens in cases of co-infections with *Borrelia burgdorferi, Anaplasma phagocytophilum, Bartonella spp. and Babesia microti*	2012	Poland	Blood donors as control group	2008	32	*Borrelia burgdorferi,* *Anaplasma phagocytophilum,* *Bartonella* spp*., Babesia microti*	Antibody (*B. burgdorferi*: IgM and IgG, *A. phagocytophilum*, *B. microti*, *Bartonella* spp.: IgG)	*B. burgdorferi*: 12.5 %; *Bartonella* spp.: 37.5 %; *A. phagocytophilum*: 9.4 %; *B. microti*: 9 %	No		43
15	Chmielewski et al.	Tick-borne pathogens *Bartonella spp., Borrelia burgdorferi sensu lato, Coxiella burnetii* and *Rickettsia spp.* may trigger endocarditis	2019	Poland	Blood donors as control group	n/a	101	*Bartonella* spp.*, Borrelia burgdorferi sensu lato,* *Coxiella burnetii, Rickettsia* spp.	Antibody (IgG)	*Bartonella henselae*: 1 %; *B. burgdorferi*: 5.8 %; *Coxiella burnetii* and *Rickettsia* spp. 0 %	No		44
16	Chmielewski et al.	Presence of *Bartonella spp*. in Various Human Populations	2007	Poland	Blood donors as control group	n/a	50	*Bartonella henselae, Bartonella quintana*	Antibody (IgG)	*B. henselae*: 5.1 %; *B. quintana*: 0 %	No		45
17	Chochlakis et al.	A serosurvey of *Anaplasma phagocytophilum* in blood donors in Crete, Greece	2008	Greece	Blood donors only	2005–2006	496	*Anaplasma phagocytophilum*	Antibody (IgG)	21.40 %	No		46
18	Cisak et al.	Risk of tick-borne bacterial diseases among workers of Roztocze National Park (south-eastern Poland)	2005	Poland	Blood donors as control group	n/a	56	*Borrelia burgdorferi, Anaplasma phagocytophilum*	Antibody (B*. burgdorferi*: IgM and IgG, *A. phagocytophilum*: IgG)	*B. burgdorferi*: 7.1 % (IgM and/or IgG), *A. phagocytophilum*: 5.4 %	No	Population: healthy male blood donors.	47
19	Coroian et al.	Seroprevalence Rates against *West Nile, Usutu,* and *Tick-Borne Encephalitis Viruses* in Blood-Donors from North-Western Romania	2022	Romania	Blood donors only	2019–2020	1200	*TBEV*	Antibody (IgG)	3.70 %	No	Low number of TBE-vaccinated individuals in the study group expected due to the timing of the study in relation to the approval of the vaccine.	48
20	De Keukeleire et al.	Seroprevalence of *Borrelia burgdorferi, Anaplasma phagocytophilum,* and *Francisella tularensis* Infections in Belgium: Results of Three Population-Based Samples	2017	Belgium	Blood donors as control group	2011	209 (rural), 193 (urban)	*Borrelia burgdorferi,* *Anaplasma phagocytophilum, Francisella tularensis*	Antibody (IgG)	*B. burgdorferi*: urban population 2.6 %, rural 2.9 %; *A. phagocytophilum*: urban population 14.5 %, rural 17.2 %; *F. tularensis*: urban population 0.5 %, rural 0.5 %	No	Population: 2 groups of blood donors: one rural, one urban.	49
21	Di Renzi et al.	Risk of acquiring tick-borne infections in forestry workers from Lazio, Italy	2010	Italy	Blood donors as control group	2008	282	*Borrelia burgdorferi, TBEV*	Antibody (IgM and IgG)	3.2 % (IgG), 7.1 % (IgM)	No	Population: blood donors from the same area as study group, free of signs and symptoms of LB and TBEV infection. No information on TBE vaccination status.	50
22	Elfving et al.	Seroprevalence of *Rickettsia* spp. infection among tick-bitten patients and blood donors in Sweden	2008	Sweden	Blood donors as control group	2002–2006	161	*Rickettsia helvetica*	Antibody (IgG)	0.60 %	No		51
23	Euringer et al.	*Tick-Borne Encephalitis Virus* IgG antibody surveillance: vaccination- and infection-induced seroprevalences, south-western Germany, 2021	2023	Germany	Blood donors only	2021	2220	*TBEV*	Antibody (IgG)	5,6 %	No	Measurement of infection-induced antibodies using NS1 IgG ELISA.	52
24	Grzeszczuk et al.	Human anaplasmosis in north-eastern Poland: Seroprevalence in humans and prevalence in Ixodes ricinus ticks	2004	Poland	Blood donors as control group	1999–2000	50	*Anaplasma phagocytophilum*	Antibody (IgG)	2 %	No	Population: age-matched blood donors, denying tick bites.	53
25	Gynthersen et al.	*Neoehrlichia mikurensis* is uncommon in rheumatological patients receiving tumour necrosis factor inhibitors and in blood donors: a retrospective cohort study	2024	Denmark	Blood donors as control group	2015–2022	400	*Neoehrlichia mikurensis*	PCR	0 %	No		54
26	Gynthersen et al.	*Neoehrlichia mikurensis* in Danish immunocompromised patients: a retrospective cohort study	2023	Denmark	Blood donors as control group	2016–2019	192	*Neoehrlichia mikurensis, Borrelia burgdorferi*	*Neoehrlichia mikurensis*: PCR; *B. burgdorfer*i: Antibody (IgG)	*Neoehrlichia mikurensis*: 0 %; *B. burgdorferi*: 5.7 %	No		55
27	Hildebrandt et al.	First confirmed autochthonous case of human *Babesia microti* infection in Europe	2007	Germany	Blood donors only	2006	44	*Babesia microti*	Antibody (IgM and IgG) and PCR	2.27 % (IgG)	Yes	Population: donors of the blood products the patient received. Almost all individuals had potential exposures to ticks, only 2 remembered previous tick infestation. 1 of 44 donors had positive antibodies, resulting in a seroprevalence of the donors' population of 2.27 %.	26
28	Hjetland et al.	Seroprevalence of antibodies to *Borrelia burgdorferi sensu lato* in healthy adults from western Norway: Risk factors and methodological aspects	2014	Norway	Blood donors only	2010	1213	*Borrelia burgdorferi*	Antibody (IgM and IgG)	9.6 % (IgG), 8.2 % (IgM)	No		56
29	Hjetland et al.	Seroprevalence of antibodies to *Tick-Borne Encephalitis Virus* and A*naplasma phagocytophilum* in healthy adults from western Norway	2015	Norway	Blood donors only	2010	1213	*TBEV, Anaplasma phagocytophilum*	Antibody (IgG)	*TBEV*: 0.4 %; *A. phagocytophilum*: 16.2 %	No	Of the five individuals with positive *TBEV* IgG, four had received vaccinations known to induce antibodies reacting in assays for *TBEV*-antibodies and one was negative for neutralizing antibodies to *TBEV*.	57
30	Hvidsten et al.	Blood donor *Borrelia burgdorferi sensu lato* seroprevalence and history of tick bites at a northern limit of the vector distribution	2017	Norway	Blood donors only	2012–2013	1567	*Borrelia burgdorferi*	Antibody (IgG)	3.38 %	No	Population: healthy blood donors in geographically extreme regions. Samples collected from 14 blood banks from 4 counties.	58
31	Jensen et al.	Evaluation of factors influencing tick bites and tick-borne infections: a longitudinal study	2021	Denmark	Blood donors only	2018–2019	344	*Borrelia burgdorferi, Rickettsia helvetica, Rickettsia felis*	Antibody (IgM and IgG)	2018: *B. burgdorferi* 7.48 % (IgM), 4.67 % (IgG); 2019: B. burgdorferi 10 % (IgM), 10 % (IgG); *Rickettisa* spp.: 0 % (1 case of seroconversion)	No		59
32	Jensen et al.	Rickettsiosis in Denmark: A nation-wide survey	2023	Denmark	Blood donors as control group	2017	181	*Rickettsia helvetica*	PCR	0 %	No		60
33	Johansson et al.	Significant variations in the seroprevalence of C6 ELISA antibodies in a highly endemic area for Lyme borreliosis: evaluation of age, sex and seasonal differences	2017	Sweden	Blood donors only	2011, 2014	273 (2011), 300 (2014)/total 573	*Borrelia* spp.	Antibody (IgM and IgG)	2011: 22 %; 2014: 24 %	No		61
34	Jovanovic et al.	Seroprevalence of *Borrelia burgdorferi* in occupationally exposed persons in the Belgrade area, Serbia	2015	Serbia	Blood donors as control group	n/a	35	*Borrelia burgdorferi*	Antibody (IgM and IgG)	8.57 %	No	Population: healthy blood donors living in the city center with no risk factors for TBI, no history of tick bites, no clinical symptoms.	62
35	Kaczmarek et al.	Asymptomatic carrier of *Babesia* spp. among blood donors - epidemiological situation in Poland	2023	Poland	Blood donors only	2022	1067	*Babesia microti, B. divergens, B. venatorum*	PCR	0 %	No		63
36	Kalmár et al.	Seroprevalence of antibodies against *Borrelia burgdorferi sensu lato* in healthy blood donors in Romania: an update	2021	Romania	Blood donors only	2019–2020	1200	*Borrelia burgdorferi*	Antibody (IgM and IgG)	ELISA: 20 % (IgM and IgG); Western Blot: 2.3 % (IgG), 1.85 % (IgM)	No		64
37	Koetsveld et al.	Serological and molecular evidence for *Spotted Fever Group Rickettsia* and *Borrelia burgdorferi sensu lato* co-infections in The Netherlands	2016	Netherlands	Blood donors as control group	n/a	150	*Borrelia burgdorferi, Rickettsia* spp.	Antibody (*B. burgdorferi*: IgM and/or IgG, *Rickettsia* spp.: IgG)	*B. burgdorferi*: 6 %; *Rickettsia* spp.: 0 %	No		65
38	Labbé Sandelin et al.	Detection of *Neoehrlichia mikurensis* DNA in blood donors in southeastern Sweden	2022	Sweden	Blood donors only	2019, 2021	1006	*Neoehrlichia mikurensis*	PCR	0.70 %	No		66
39	Larsen et al.	Detection of specific IgG antibodies in blood donors and *Tick-Borne Encephalitis Virus* in ticks within a non-endemic area in southeast Norway	2014	Norway	Blood donors only	2012	461	*TBEV*	Antibody (IgG)	0.65 % (only non-vaccinated), 1.73 % (overall incl. vaccinated)	No	Population: samples from 6 geographically different locations. Seroprevalence rates depended on vaccination status.	67
40	Lindblom et al.	Seroreactivity for *Spotted Fever Rickettsiae* and co-infections with other tick-borne agents among habitants in central and southern Sweden	2013	Sweden	Blood donors as control group	2012	80	*Rickettsia* spp.	Antibody (IgM and IgG)	1.25 %	No	Population: blood donors with no clinical symptoms of infection.	68
41	Łysakowska et al.	The seroprevalence of *Bartonella* spp. in the blood of patients with musculoskeletal complaints and blood donors, Poland: a pilot study	2019	Poland	Blood donors as control group	n/a	65	*Bartonella* spp.	Antibody (IgM and IgG)	B. henselae: 30.8 % (IgG), 3.1 % (IgM), B. quintana: 4.6 % (IgG), 1.54 % (IgM)	No	Population: blood donors with no musculoscelettal symptoms.	69
42	Magyar et al.	New geographical area on the map of *Crimean-Congo Hemorrhagic Fever Virus*: First serological evidence in the Hungarian population	2021	Hungary	Blood donors only	2008–2017	2700	*CCHFV*	Antibody (IgG)	0.44 %	No		70
43	Marvik et al.	Low prevalence of *Tick-Borne Encephalitis Virus* antibodies in Norwegian blood donors	2021	Norway	Blood donors only	2019	1123	*TBEV*	Antibody (IgG)	0.4 % (non-vaccinated), 1.9 % (overall including vaccinated)	No		71
44	Monsalve Arteaga et al.	*Crimean-Congo hemorrhagic fever (CCHF) virus*-specific antibody detection in blood donors, Castile-León, Spain, summer 2017 and 2018	2020	Spain	Blood donors only	2017–2018	516	*CCHFV*	Antibody (IgG)	0.58–1.16 %	No	Seroprevalence rates depended on diagnostic assay.	72
45	Müller et al.	Detection of *Bartonella* spp. in Ixodes ricinus ticks and *Bartonella* seroprevalence in human populations	2016	Austria	Blood donors as control group	2005	100	*Bartonella* spp.	Antibody (IgG)	B. quintana: 22 %, B. henslae: 1 %, both: 5 %	No		73
46	Munro et al.	Seroprevalence of Lyme borreliosis in Scottish blood donors	2015	Scotland	Blood donors only	2010–2011	1440	*Borrelia burgdorferi*	Antibody (IgG)	4.20 %	No		74
47	Mygland et al.	Chronic polyneuropathy and Lyme disease	2006	Norway	Blood donors as control group	1994–2004	247	*Borrelia burgdorferi*	Antibody (IgG)	18 %	No	IgM was also measured, but not reported.	75
48	Ocias et al.	Evidence of *Rickettsiae* in Danish patients tested for Lyme neuroborreliosis: a retrospective study of archival samples	2018	Denmark	Blood donors as control group	2011–2015	171	*Rickettsia* spp.	Antibody (IgM and IgG) and PCR	30 % (antibodies), 0 % (PCR)	No		76
49	Pawełczyk et al.	Seroprevalence of six pathogens transmitted by the Ixodes ricinus ticks in asymptomatic individuals with HIV infection and in blood donors	2019	Poland	Blood donors as control group	n/a	199	*Borrelia burgdorferi s.l., Anaplasma phagocytophilum, Ehrlichia* spp*., Babesia* spp*., Rickettsia* spp*., Bartonella henselae*	Antibody (IgM and IgG)	13.1 % (IgM), 5.0 % (IgG)	No		77
50	Santos et al.	Human exposure to *Anaplasma phagocytophilum* in Portugal	2006	Portugal	Blood donors as control group	2002	96	*Anaplasma phagocytophilum*	Antibody (not specified)	1 % (confirmed cases), 3.1 % (possible)	No		78
51	Sonnleitner et al.	*Spotted Fever Group-Rickettsiae* in the Tyrols: evidence by seroepidemiology and PCR	2013	Austria	Blood donors only	2009	1634	*Rickettsiae* spp.	Antibody (IgG)	R. helvetica: 10.6 % (North Tyrol), 7.4 % (South Tyrol), R. honei: 5.5 % (North Tyrol), 4.5 % (South Tyrol)	No	Population: healthy blood donors representing the total population of the study area regarding gender, profession and altitude of residency.	79
52	Sonnleitner et al.	Human seroprevalence against *Borrelia burgdorferi sensu lato* in two comparable regions of the eastern Alps is not correlated to vector infection rates	2015	Austria	Blood donors only	2009	1607	*Borrelia burgdorferi*	Antibody (IgG)	7.2 % (North Tyrol), 1.5 % (South Tyrol)	No	Population: healthy blood donors representing the population of the study area regarding gender, proffession, altitude of residence.	80
53	Sonnleitner et al.	Risk assessment of transfusion-associated Babesiosis in Tyrol: appraisal by seroepidemiology and polymerase chain reaction	2014	Austria	Blood donors only	2009	988	*Babesia* spp.	Antibody (IgG)	B. divergens: 2.1 %, B. microti 0.61 %	No	Population: healthy blood donors representing the total population of the study area regarding sex, profession, and altitude of residency.	81
54	Stańczak et al.	Kampinos National Park: a risk area for *Spotted Fever Group Rickettsioses*, central Poland?	2016	Poland	Blood donors as control group	2012–2013	30	*SFG rickettsiae*	Antibody (IgG)	13.30 %	No	Population: blood donors who denied a tick bite 6 months prior to the investigation.	82
55	Tomasiewicz et al.	The Risk of Exposure to *Anaplasma phagocytophilum* Infection in Mid-Eastern Poland	2004	Poland	Blood donors as control group	n/a	30	*Anaplasma phagocytophilum*	Antibody (IgG)	0 %	No	Population: healthy blood donors who denied tick bites.	83
56	Walder et al.	Serological evidence for Human Granulocytic Ehrlichiosis in Western Austria	2003	Austria	Blood donors only	2001	357	*Anaplasma phagocytophilum, Borrelia burgdorferi*	Antibody (IgM and IgG)	A. phagocytophilum: 9 %, B. burgdorferi: 8.4 %	No	Population: healthy blood donors who together represent the current demographic situation in Tyrol (gender, residence).	84
57	Zákutná et al.	Pilot Cross-Sectional Study of Three Zoonoses (Lyme Disease, Tularaemia, Leptospirosis) among Healthy Blood Donors in Eastern Slovakia	2015	Slovakia	Blood donors only	2011	124	*Borrelia burgdorferi, Francisella tularensis*	Antibody (IgG)	B. burgdorferi: 15 % (ELPAGA), 1.6 % (Western Blot IgG), F. tularensis 4 % (ELPAGA), 0.8 % (Western Blot IgG)	No	Seroprevalence rates depended on diagnostic assay.	85

### 3.2 Tick-borne pathogens and seroprevalences

**Fig. 3:**
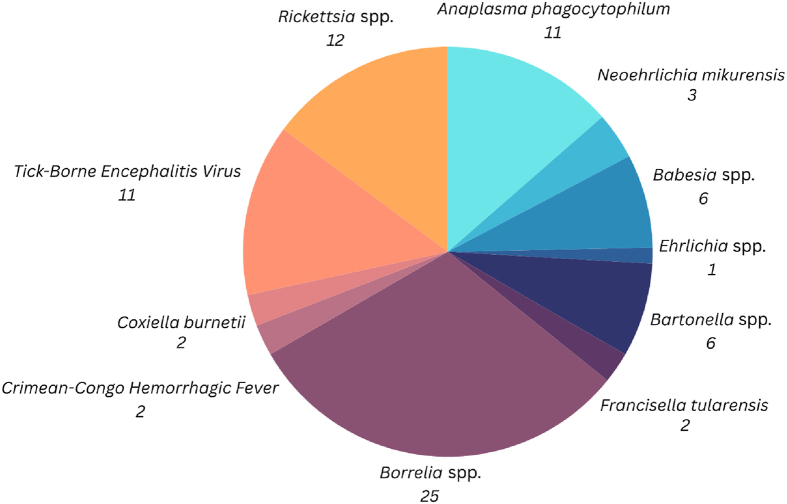
Overall Number of Tick-borne Pathogens examined in Sera of Blood Donors in Europe.

The included studies examined the exposure to 11 tick-borne pathogens in the blood donors’ sera overall (Fig. 3). The most studied pathogens were *Borrelia* spp. (25 studies), followed by *Rickettsia* spp. (12 studies), *TBEV* and *Anaplasma phagocytophilum* (11 studies each), *Babesia* spp. and *Bartonella* spp. (6 studies each).

Most studies (48 studies) used antibody detection as diagnostic technique to identify the seroprevalences, whereas 5 studies conducted Polymerase Chain Reaction (PCR) testing, and 4 studies used both techniques.

Table 1, Table 2 summarize the seroprevalence rates. The highest seroprevalence rates were observed for *TBEV*, *Bartonella* spp., *Rickettsia* spp. and *Borrelia* spp.; however, the rates varied across individual studies. The highest overall seroprevalence was observed for *TBEV* in Switzerland with a rate of 81.1 %, and here it must be stressed that this was a cohort of vaccinated individuals (Table 3). In Poland, a study found that 37.5 % of participants had antibodies against *Bartonella* spp. Meanwhile, a study in Greece reported a seroprevalence rate of 21.4 % for *Anaplasma phagocytophilum*. The highest seropositivity rate for *Borrelia* spp. was recorded in Sweden, at 24 %. For *Rickettsia* spp., the highest seropositivity rate was 30 %, observed in Danish patients who were initially tested for neuroborreliosis.

**Table 2:** Seroprevalence Rates of Tick-borne Pathogens examined in Sera of Blood Donors in Europe. ∗Note: Seroprevalences including vaccinated individuals.

Country	Examined pathogen	Found seroprevalence(s)	Diagnostic technique	Reference
Austria	*Anaplasma phagocytophilum*	9 %	Antibody (IgM and IgG)	84
	*Babesia* spp.	0 %	PCR	37
		*B. divergens*: 2.1 %, *B. microti* 0.61 %	Antibody (IgG)	81
	*Bartonella* spp.	*B. quintana*: 22 %, *B. henslae*: 1 %, both: 5 %	Antibody (IgG)	73
	*Borrelia* spp.	*B. burgdorferi*: 8.4 %	Antibody (IgM and IgG)	84
		*B. burgdorferi*: 7.2 % (North Tyrol), 1.5 % (South Tyrol)	Antibody (IgG)	80
	*Rickettsiae* spp.	*R. helvetica*: 10.6 % (North Tyrol), 7.4 % (South Tyrol), *R. honei*: 5.5 % (North Tyrol), 4.5 % (South Tyrol)	Antibody (IgG)	79
Belgium	*Borrelia* spp.	*B. burgdorferi, B. afzelii, B. garinii*: 2 %	Antibody (IgM and IgG)	41
		*B. burgdorferi*: 2.6 % (urban population), 2.9 % (rural population)	Antibody (IgG)	49
	*Anaplasma phagocytophilum*	14.5 % (urban population), 17.2 % (rural population)	Antibody (IgG)	49
	*Francisella tularensis*	0.5 % (urban population), 0.5 % (rural population)	Antibody (IgG)	49
Denmark	*Borrelia* spp.	*B. burgdorferi:* 5.7 %	Antibody (IgG)	55
		*B. burgdorferi:* 2018: 7.48 % (IgM), 4.67 % (IgG); 2019: 10 % (IgM), 10 % (IgG)	Antibody (IgM and IgG)	59
	*Neoehrlichia mikurensis*	0 %	PCR	54
		0 %	PCR	55
	*Rickettsia* spp.	*R. helvetica, R. felis*.: 0 % (1 case of seroconversion)	Antibody (IgM and IgG)	59
		*R. helvetica*: 0 %	PCR	60
		30 % (antibodies), 0 % (PCR)	Antibody (IgM and IgG) and PCR	76
France	*Rickettsia* spp.	*R. conorii*: 1 %; *R. typhi*: 0.2 %; *R. akari*: 0 %; *R. felis*: 0.6 %	Antibody (IgG)	40
Germany	*TBEV*	57 % (overall including vaccinated); 5.6 % (presumably infection-induced)∗	Antibody (IgG)	52
	*Babesia microti*	2.27 % (IgG)	Antibody (IgM and IgG) and PCR	26
Greece	*Anaplasma phagocytophilum*	21.40 %	Antibody (IgG)	46
Hungary	*CCHFV*	0.44 %	Antibody (IgG)	70
Italy	*Borrelia* spp.	*B. burgdorferi*: IgG 3.2 %, IgM 7.1 %	Antibody (IgM and IgG)	50
	*TBEV*	0.0 %	Antibody (IgM and IgG)	50
Netherlands	*Borrelia* spp.	*B. burgdorferi:* 6 %	Antibody (IgM and/or IgG)	65
	*Rickettsia* spp.	0 %	Antibody (IgG)	65
Norway	*Anaplasma phagocytophilum*	16 %	Antibody (IgG)	57
	*Borrelia* spp.	*B. burgdorferi:* 8.2 % (IgM), 9.6 % (IgG)	Antibody (IgM and IgG)	56
		*B. burgdorferi:* 18 %	Antibody (IgG)	75
		*B. burgdorferi:* 3.38 %	Antibody (IgG)	58
	*TBEV*	0.65 % (non-vaccinated), 1.73 % (overall, including vaccinated)∗	Antibody (IgG)	67
		0.4 % (non-vaccinated), 1.9 % (overall, including vaccinated)∗	Antibody (IgG)	71
		0.4 %∗	Antibody (IgG)	57
Poland	*Anaplasma phagocytophilum*	0 %	Antibody (IgG)	83
		2 %	Antibody (IgG)	53
		3.0 % (IgM), 1.5 % (IgG)	Antibody (IgM and IgG)	77
		9.40 %	Antibody (IgG)	43
		5.40 %	Antibody (IgG)	47
		0 %	PCR	39
	*Babesia* spp.	1.0 % (IgM), 1.5 % (IgG)	Antibody (IgM and IgG)	77
		*B. microti, B. divergens, B. venatorum*: 0 %	PCR	63
		*B. microti*: 9 %	Antibody (IgG)	43
	*Bartonella* spp.	37.50 %	Antibody (IgG)	43
		1 %	Antibody (IgG)	44
		*B. henselae*: 5.1 %; *B. quintana*: 0 %	Antibody (IgG)	45
		*B. henselae*: 30.8 % (IgG), 3.1 % (IgM), *B. quintana*: 4.6 % (IgG), 1.54 % (IgM)	Antibody (IgM and IgG)	69
		*B. henslae:* 4.5 % (IgM), 4.5 % (IgG)	Antibody (IgM and IgG)	77
	*Borrelia* spp.	*B. burgdorferi*: 0 %	Antibody (IgG)	39
		*B. burgdorferi*: 12.5 %	Antibody (IgM and IgG)	43
		*B. burgdorferi:* 7.1 % (IgM and/or IgG)	Antibody (IgM and IgG)	47
		*B. burgdorferi sensu lato*: 5.8 %	Antibody (IgG)	44
		*B. burgdorferi sensu lato:* 13.1 % (IgM), 5.0 % (IgG)	Antibody (IgM and IgG)	77
	*Coxiella burnetii*	0 %	Antibody (IgG)	38
		0 %	Antibody (IgG)	44
	*Ehrlichia* spp.	4.5 % (IgM), 2.5 % (IgG)	Antibody (IgM and IgG)	77
	*Rickettsia* spp.	0 %	Antibody (IgG)	44
		0 %	Antibody (IgG)	39
		*Spotted Fever Group Rickettsia:* 1.0 % (IgG)	Antibody (IgM and IgG)	77
		*Spotted Fever Group Rickettsiae:* 13.3 %	Antibody (IgG)	82
	*TBEV*	0 %	Antibody (IgG)	39
Portugal	*Anaplasma phagocytophilum*	1 % (confirmed cases), 3.1 % (possible)	Antibody (not specified)	78
Romania	*Borrelia* spp.	*B. burgdorferi*: ELISA: 20 % (IgM and IgG); Western Blot: 2.3 % (IgG), 1.85 % (IgM)	Antibody (IgM and IgG)	64
	*TBEV*	3.70 %	Antibody (IgG)	48
Scotland	*Borrelia* spp.	*B. burgdorferi:* 4.2 %	Antibody (IgG)	74
Serbia	*Borrelia* spp.	*B. afzelii:* 6.67 %	Antibody (IgG)	33
		*B. burgdorferi:* 8.57 %	Antibody (IgM and IgG)	62
	*TBEV*	4 %	Antibody (IgG)	34
Slovakia	*Borrelia* spp.	*B. burgdorferi:* 4.4 %–15.6 % (cumulatively 12.8 %)	Antibody (IgG)	36
		*B. burgdorferi:* 15 % (ELPAGA), 1.6 % (Western Blot IgG)	Antibody (IgG)	85
	*Francisella tularensis*	4 % (ELPAGA), 0.8 % (Western Blot IgG)	Antibody (IgG)	85
Spain	*Borrelia* spp.	*B. burgdorferi:* 5.1 %	Antibody (IgG)	35
	*CCHFV*	0.58–1.16 %	Antibody (IgG)	72
Sweden	*Borrelia* spp.	*Borrelia afzelii, B. garinii:* 8 % (individuals with no previous history of Lyme Borreliosis), 12 % (with previous Lyme Borreliosis); 1 % (undetermined)	Antibody (IgG)	42
		2011: 22 %, 2014: 24 %	Antibody (IgM and IgG)	61
	*Neoehrlichia mikurensis*	0.70 %	PCR	66
	*Rickettsia* spp.	*R. helvetica:* 0.6 %	Antibody (IgG)	51
		1.25 %	Antibody (IgM and IgG)	68
	*TBEV*	27.50 % (vaccinated 25.1 %, presumably infected 2.4 %)∗	Antibody (IgG)	32
Switzerland	*TBEV*	23.7–53.3 %∗	Antibody (IgG)	30
		Individuals with previous TBEV infection: 0.34 %, thereof 59.4 % seropositive; 81.1 % (vaccinated individuals); 5.6 % (non-vaccinated individuals)∗	Antibody (IgG)	31

### 3.3 Transfusion-transmitted infections

Of the 57 studies that met our inclusion criteria, only one examined a transfusion-transmitted infection with *Babesia microti*.

### 3.4 Risk of bias assessment

After assessment using the JBI Critical Appraisal Checklist for Studies Reporting Prevalence Data, all studies were appraised for inclusion (Supplementary File 3).

## 4 Discussion

In this systematic review, 57 studies conducted in 19 European countries were included, examining the serological and molecular evidence of 11 tick-borne infections, the majority of which were diagnosed through antibody testing. Heterogeneity was observed in terms of population, study design, exposure, and pathogen.

Several systematic reviews have investigated the seroprevalence of TBIs across different populations and regions. For example, high-risk groups exposed to tick habitats in their occupational settings, such as forestry and agricultural workers, animal handlers and farmers, have been studied, revealing an increased risk of TBIs, namely of LB and CCHF among certain professional subgroups [86,87]. However, comprehensive research specifically focused on the topic of seroprevalence of TBIs in European blood donors has not yet been conducted.

The seroprevalence rates found in this study indicate a significant level of exposure to TBIs in the general population, demanding greater attention and further improvements of public health measures to reduce the risk of infection. Considering that the population examined consisted of blood donors, the concern arises whether this poses a risk for blood sample recipients. Both these topics are linked and deserve discussion:

### 4.1 TBI prevention in the general population

TBIs result in a significant economic burden [88], which continues to rise [89]. For instance, tick bites and LB resulted in societal cost of €19.3 million in the Netherlands in 2010 [90], while TBE and its consequences cost Russia $49.5 million in 2011 [91]. Despite these significant economic impacts, awareness of these infections and the availability of vaccines remains lower than for other diseases, particularly in non-endemic regions [92].

Vaccination is an effective preventive measure. While no specific therapy for TBE exists, two vaccines are licensed and available in Europe [92], both of which have demonstrated excellent field effectiveness, reducing the risk of TBE in vaccinated individuals compared to non-vaccinated patients across all age groups [93]. Although the ongoing development of new vaccines against LB is promising, public acceptance and compliance remain uncertain, primarily due to the anticipated complexity of the vaccination schedule [94]. Against other TBIs, efforts to develop vaccines have not yet been successful. A prominent example is CCHF, a widespread and sometimes fatal disease. Despite the large number of potential vaccine candidates, there are no approved vaccines so far [95,96].

Bacterial and parasitical TBIs, such as LB, Human Granulocytic Anaplasmosis, and Babesiosis, can be effectively managed with appropriate antimicrobial therapy [97]. In contrast, viral TBIs usually lack specific antiviral treatments, and the management of these infections primarily relies on supportive care. The antiviral drug ribavirin has been explored as a treatment for CCHF, with studies suggesting it could reduce mortality when administered early [98]. However, systematic reviews have mostly been unable to conclusively prove its therapeutic benefit [[99], [100], [101]]. The evidence remains inconclusive, and the use of ribavirin for CCHF is still debated.

As long as vaccines remain unavailable for some TBIs, treatment options are limited for others, and measures to control tick populations are inadequate, TBI control must focus on preventive strategies to avoid tick bites [102]. While complete avoidance of tick habitats by the general population may not be practical, increasing public awareness of the health risks posed by ticks and the importance of personal protection measures likely represents the most effective prevention currently available [103]. Personal protection measures include the use of protective clothing and tick repellents [104]. However, some of the most commonly used repellents like N,N-Diethyl-meta-toluamide (DEET), require high concentrations to repel ticks [105]. Additionally, there is evidence that insecticide-treated clothing can provide protection against arthropod bites [106].

Furthermore, developers are creating technological tools such as smartphone applications to help increase public knowledge of ticks and the use of preventive measures [102]. Also, tools for new monitoring strategies have been developed, such as antigenic testing of TBIs through salivary proteins (in ticks), which could be an easy and useful epidemiological tool for improved vector surveillance [107].

### 4.2 Blood donors and risk of transfusion-transmitted infections

The seroprevalence rates observed in this study were relatively high, particularly considering that the population examined consisted of blood donors. Beyond the inferred exposure from these findings, the key question arises whether this poses a risk for blood recipients. Most of the studies included in this review assessed seroprevalence using antibody testing (IgM and/or IgG). As the immune system produces antibodies in response to infection, elevated antibody levels indicate current or prior exposure to a pathogen but do not provide direct evidence on the infectivity of blood samples. PCR testing, which detects the presence of a pathogen's genetic material, can confirm the pathogen's presence in the body and suggest potential infectivity. However, PCR tests do not directly measure infectivity, as they can also detect genetic material from non-viable pathogens. Furthermore, except for quantitative PCR (qPCR), these tests do not quantify the infectious load. Only a small subset of the studies included utilized PCR testing.

This study highlights significant exposure of blood donors to TBIs across various regions of Europe.

While *Babesia* spp. is known to be transmissible via blood transfusion, it remains unclear which other TBIs could be similarly transmitted. Nevertheless, there is potential for other tick-borne pathogens, such as *Borrelia burgdorferi*, which is widely prevalent in Europe, to cause transfusion-transmitted infections [25]. This raises important questions about the feasibility and utility of screening blood samples for TBIs before transfusion.

According to the European Committee on Blood Transfusion, all donations reported by the member states (MS) are tested for *HIV* antibodies (anti-HIV 1/2), *Hepatitis B Virus* surface antigen (HBsAG) and *Hepatitis C Virus* antibodies (anti-HCV). Additionally, 96 % of the donations are tested for syphilis, while *Human T-Lymphotropic Virus* antibodies (anti-HTLV 1/2) testing is performed in only 20 % of the MS. Switzerland has been conducting *Hepatitis E Virus* screening tests since 2018, making it the only MS applying such tests to all blood donations [108].

Based on the blood donor's travel history, the Swiss Blood Donation Service may perform serological testing of the donated serum for *Tryopanosoma cruzi* (the causative agent of Chagas disease), *Plasmodium* spp. (responsible for malaria), and *Cytomegalovirus*. Additionally, PCR testing may be conducted for *West Nile Virus*. Donations intended for plasma fractionation will also be tested for *Parvovirus B19* and *Hepatitis A Virus*. Donations are not routinely screened for TBIs. However, the Swiss Blood Donation Service will reject a donation if the donor indicates on the pre-donation questionnaire that they were bitten by a tick within the last four weeks [109]. According to information provided by the Swiss Blood Donation Service, regional blood donation services hold regular meetings to discuss current epidemiological trends. While TBIs are considered a relevant issue, the primary focus regarding emerging infections in donated blood is currently on arboviruses. At present, deferring blood donations based on a donor's report of a tick bite within the last four weeks is considered sufficient. However, studies have suggested that self-reported bites may not be reliable indicators of serologic status of blood donors [110].

Given that blood donors must typically be asymptomatic of any type of infection at the time of donation, one approach to identifying pauci- or asymptomatic carriers would be to implement universal screening of all donated samples. However, the broad screening for TBIs necessitates careful consideration of cost-effectiveness; as the cost of a PCR test for *Borrelia burgdorferi*, *TBEV* or *Babesia microti* in Switzerland currently stands at CHF 119 (approx. €126) per test [111]. An alternative strategy could involve assessing each donor's individual risk for exposure to tick-borne pathogens, such as through questionnaires addressing potential occupational or recreational exposure, geographic location, travel history, and donor origin. This approach could enable targeted screening, particularly in endemic regions. On the official blood donation questionnaires in Switzerland, Germany, Austria and Luxembourg for example, donors are only required to answer whether they have had a tick bite in the last four or eight weeks [109,[112], [113], [114]]. However, no additional evaluation is carried out to assess an individual's risk of tick exposure.

In the United States, more than 200 cases of transfusion-transmitted Babesiosis (TTB) have been reported since 1980. In 2019, the Food and Drug Administration (FDA) has licensed two nucleic acid amplification tests (NAATs) for the detection of *Babesia* spp. Since 2020, these NAATs have been used to screen blood donors in high-risk states to reduce the risk of TTB. Screening for *Babesia* spp. was the first test to be implemented regionally due to the pathogen's limited geographic distribution, which initially made test development less appealing for manufacturers and complicated its implementation. The FDA's determination that Babesiosis is a relevant transfusion-transmitted infection was based on several factors, including the severity of the disease, the confirmed transmission through blood transfusions, and the availability of appropriate screening tests. The incidence and prevalence of *Babesia* spp. infections in potential donor populations, particularly in high-risk states, further justified the need for implementing screening protocols to mitigate the risk of TTB [115]. Studies assessing the impact of this initiative have shown a significant reduction of TTB cases in these areas since the implementation, demonstrating the high effectiveness of this testing [116]. Although rare, TTB remains a possibility despite regional screening effort [117]. Cost analysis studies conducted in the United States prior to the implementation of TTB screening showed no consensus on cost-effectiveness of such measures [[118], [119], [120]]. Following the FDA's recommendation to implement donor screening, the cost-effectiveness has yet to be evaluated. A pathogen reduction device has not yet been licensed [121].

In some other regions, *Babesia* spp. screenings have been evaluated, but not yet implemented. Risk-based decision making processes have been used to address identified risks in blood transfusion practice and blood system policy development [122]. For example, in Canada, the likelihood of clinically relevant TTB was assessed to be low, suggesting that testing would have little utility [123].

For other tick-borne pathogens, systematic NAAT screenings for blood donors have not been implemented up to this date.

The significance of co-infections in TBIs has been well-documented, with such co-infections being associated with increased morbidity in affected patients [21]. Therefore, when screening for a single TBI, it is crucial to consider the potential for co-infections, as they may significantly impact patient outcomes.

In addition to pre-transfusion screening and individual risk assessment, clinicians should conduct thorough post-transfusion follow-ups to identify potential TTTBIs, evaluate the differential diagnosis of suspected transfusion-associated infections, and test for a broad spectrum of tick-borne pathogens in cases where TTTBIs are suspected.

In addition to advancements in public health and epidemiology, clinicians in both practices and hospitals should remain vigilant about the wide range of pathogens transmitted by ticks. It is advisable to consider testing for various TBIs beyond *Borrelia* spp. in patients presenting with nonspecific systemic symptoms following a tick bite. Furthermore, new tick borne pathogens continue to be discovered, such as the recently detected *Alongshan* virus in Swiss *Ixodes ricinus* ticks. However, it remains unknown whether the virus can be transmitted to humans and to what extent this poses a significant public health risk [124].

Controlling TBIs is particularly challenging due to the complex transmission cycles involving various tick species, animal hosts, and changing environmental conditions. Ticks and animals serve as primary reservoirs for the pathogens responsible for TBIs. Therefore, addressing this issue requires a One Health approach, which integrates human and veterinary medicine to enhance disease management. Given that climate change is a key factor driving the spread of TBIs, efforts to mitigate its effects are also crucial. The growing threat of TBIs necessitates multidisciplinary strategies to reduce their impact on public health, making the adoption of a One Health approach essential for improving the diagnosis, treatment, and prevention of these diseases [125].

### 4.3 Strengths and limitations

This systematic review offers a comprehensive synthesis of the existing literature on seroprevalence of TBIs in blood donors across Europe, providing an overview of the current state of knowledge by infection and geography. The systematic approach, incorporating a comprehensive search strategy, strict inclusion and exclusion criteria, and meticulous data extraction processes, ensures that the review is methodologically robust and reliable. The use of multiple databases minimized the risk of publication bias and ensured a wide capture of relevant studies.

One of the primary limitations is the heterogeneity across the included studies. This heterogeneity stems from variations in population characteristics, sample sizes, and diagnostic methods.

For TBE, population heterogeneity was particularly evident: in some studies, groups in which seroprevalence was measured using antibodies included vaccinated individuals, while other studies excluded such individuals (Table 3). Since vaccination can lead to elevated antibody levels, the strikingly high seroprevalence rates for TBE found in some studies should be interpreted with caution. It is not always possible to differentiate between positive antibodies due to a vaccination or a previous infection. This complicated the comparison between the individual studies as well as the comparison with studies that excluded vaccinated blood donors. Some studies did not declare whether the studied blood donors had a history of vaccination or not.

**Table 3:** Seroprevalence rates of TBEV by vaccination status.

Country	Seroprevalence VACCINATED	Seroprevalence NON-VACCINATED	Seroprevalence other/unclear	Diagnostic technique	Reference
*Germany*	57 % (overall including past vaccination)		5.6 % (measurement of TBEV NS1 IgG; presumably infection-induced)	Antibody (IgG)	52
*Italy*			0.0 %	Antibody (IgM and IgG)	50
*Norway*	1.08 %	0.65 %		Antibody (IgG)	67
	1.5 %	0.4 %		Antibody (IgG)	71
	0.4 %			Antibody (IgG)	57
*Poland*			0 %	Antibody (IgG)	39
*Romania*			3.70 % (“not expected to be vaccinated”)	Antibody (IgG)	48
*Serbia*		4 %		Antibody (IgG)	34
*Sweden*	25.1 %	2.4 %		Antibody (IgG)	32
*Switzerland*	6.4–57 % (vaccinated individuals; depending on diagnostic assay)		23.7–53.3 % (overall; including vaccinated against TBEV and/or other flaviviruses and non-vaccinated)	Antibody (IgG)	30
	81.1 %	5.6 %	0.34 % (individuals with previous TBEV infection)	Antibody (IgG)	31

Additionally, the exclusion of studies not written in English, German, French, Spanish, Italian or Finnish may have led to the omission of relevant data, contributing to language bias.

### 4.4 Possibilities for future studies

The findings of this review highlight several important directions for future research.

Given the diversity of pathogens observed, there is a need for a broader focus on a wider range of tick-borne pathogens. Expanding the scope of future studies to encompass the expanding spectrum of tick-borne pathogens will enhance our understanding of the overall epidemiological landscape and potential co-infections, which may have important implications for public health strategies and clinical management.

To evaluate the temporal and geographical epidemiological trends, more comprehensive testing would be beneficial and One Health collaborations are indicated. In Europe, the European Committee on Blood Transfusion serves as a key advisory board within the Council of Europe. It develops standards and guidelines for blood transfusion practices, focusing on safety and quality assurance, including the prevention of transfusion-transmitted infections. Through its efforts, the European Committee on Blood Transfusion fosters collaboration between European countries to effectively enhance blood transfusion practices across the region.

While seroprevalence rates using antibody testing provide valuable insight into exposure to tick-borne pathogens, they offer limited information about the potential current infectivity of the sera. Future studies should incorporate NAAT of sera to better assess the presence of pathogens and to distinguish between past exposure and ongoing risk of transmission, which is especially relevant in transfusion medicine.

## 5 Conclusions

This systematic review included 57 studies encompassing 19 European countries and 11 different TBIs. Several demonstrated significant seroprevalences of a wide range of TBIs in blood donor's sera. The highest seroprevalence rates were observed for Tick-Borne Encephalitis Virus (notably, some populations included vaccinated individuals), *Bartonella* spp., *Rickettsia* spp. and *Borrelia* spp.; however, the rates varied across individual studies. The majority of studies used antibody detection as a diagnostic technique.

This review highlights the need for future research to broaden its focus on a wider range of tick-borne pathogens to better understand the epidemiological landscape of TBIs. Additionally, incorporating NAAT of donated blood will improve the ability to differentiate between past exposure and current presence of pathogens with potential infectivity, allowing to better assess transmission risks in transfusion medicine.

## CRediT authorship contribution statement

**Sophie Mathys:** Writing – review & editing, Writing – original draft, Visualization, Methodology, Investigation, Formal analysis, Data curation, Conceptualization. **Nejla Gültekin:** Writing – review & editing. **Zeno Stanga:** Writing – review & editing. **Ismail Ülgür:** Writing – review & editing. **Patricia Schlagenhauf:** Writing – review & editing, Supervision, Project administration, Investigation, Formal analysis, Data curation, Conceptualization.

## Patient consent statement

Not applicable for this study.

## Research ethics approval

This is a systematic review of published articles. Ethical approval is not applicable.

## Data availability statement

The data that support the findings of this study are provided with the manuscript.

## Permissions to reproduce materials from other sources

Not applicable.

## Funding statement

This research did not receive any specific grant from funding agencies in the public, commercial, or not-for-profit sectors.

## Declaration of competing interest

The authors declare that they have no known competing financial interests or personal relationships that could have appeared to influence the work reported in this paper.
