# Supplementary material for: Seroprevalence of tick-borne infections in blood donors in Europe: a systematic review
Source: New Microbes New Infect. 2025 May 10;65:101597. doi: 10.1016/j.nmni.2025.101597 (PMC12149587; doi:10.1016/j.nmni.2025.101597)
Supplement: Multimedia component 1 [file mmc1.docx]

**Appendix**

**Supplementary File 1:**

**Search Strings for Seroprevalence of Tick-borne Infections for Different Databases**

EMBASE*:

('tick borne disease'/exp **OR** ('tick*' AND ('infection*' OR 'disease*' OR 'borreli*' OR 'coxiella' OR 'q fever' OR 'rickettsia*' OR 'bartonell*'))) **AND** 'europe'/exp **AND** ('epidemio*' OR 'frequency' OR 'occurrence' OR 'incidenc*' OR 'prevalence*') **AND** [2000-2024]/py **NOT** ([animals]/lim NOT [humans]/lim)

PubMed*:
("Tick-Borne Diseases"[Mesh] OR ("Tick*" AND ("infection*" OR "disease*" OR "Borreli*"[Mesh] OR "Coxiell*"[Mesh] OR "Rickettsi*"[Mesh] OR "Bartonell*"[Mesh]))) AND "Europe"[Mesh] NOT (animals [Mesh] NOT humans [Mesh]) AND (2000:2024[pdat]) AND (epidemio* OR frequency OR occurrence OR incidenc* OR prevalence*)

CINAHL*:

( ((MH "Tick-Borne Diseases+") OR (Tick* AND (infection* OR disease* OR (MH Borreli*+) OR (MH Coxiell*+) OR (MH Rickettsi*+) OR (MH "Bartonell*+")))) ) AND (MH Europe+) AND (epidemio* OR frequency OR occurrence OR incidenc* OR prevalence*) NOT ( ((MH animals+) NOT (MH humans+)) )

Limiters - Publication Date: 20000101-20241231

Scopus:
(TITLE-ABS-KEY( "tick borne*" ) OR (TITLE-ABS-KEY("tick*") AND (TITLE-ABS-KEY(("infection*") OR ("disease*") OR ("borreli*") OR ("encephalitis") OR ("rickettsia*") OR ("q fever") OR ("coxiell*"))))) AND (TITLE-ABS-KEY(( "Europe" ) OR ("Aland*" ) OR ( "Albania*" ) OR ( "Andorra" ) OR ( "Austria*" ) OR ( "Belarus*" ) OR ( "Belgi*" ) OR ( "Bosnia*" ) OR ( "Bulgaria*" ) OR ("Channel Island*" ) OR ( "Croatia*" ) OR ( "Czech*" ) OR ( "Denmark" ) OR ( "Danish" ) OR ( "Estonia*" ) OR ( "Faroe*" ) OR ( "Finland" ) OR ( "Finnish" ) OR ( "France" ) OR ( "French" ) OR ( "German*" ) OR ( "Gibraltar*" ) OR ( "Greece" ) OR ( "Greek" ) OR ( "Vatican*" ) OR ( "Hungar*" ) OR ( "Iceland*" ) OR ( "Ireland" ) OR ( "Irish" ) OR ( "Isle of Man " ) OR ( "Ital*" ) OR ( "Kosov*" ) OR ( "Latvia*" ) OR ( "Liechtenstein" ) OR ( "Lithuania*" ) OR ( "Luxembourg" ) OR ( "Malt*" ) OR ( "Monaco" ) OR ( "Montenegr*" ) OR ( "Netherlands" ) OR ( "Dutch" ) OR ( "Macedonia*" ) OR ( "Norw*" ) OR ( "Svalbard*" ) OR ( "Jan Mayen*" ) OR ( "Poland" ) OR ( "Polish" ) OR ( "Portug*" ) OR ( "Moldov*" ) OR ( "Romania*" ) OR ( "Russia*" ) OR ( "San Marino" ) OR ( "Serbia*" ) OR ( "Slovakia*" ) OR ( "Slovenia*" ) OR ( "Spain" ) OR ( "Spanish" ) OR ( "Swed*" ) OR ( "Switzerland" ) OR ( "Swiss" ) OR ( "Ukrain*" ) OR ( "Great Britain" ) OR ( "British" ) OR ("United Kingdom" ) OR ("Northern Ireland" ))) AND TITLE-ABS-KEY(("epidemio*") OR (“frequency”) OR (“occurrence”) OR (“incidenc*”) OR (“prevalence*”)) AND PUBYEAR > 1999 AND PUBYEAR < 2025 AND NOT ( ( INDEXTERMS ( animals OR animal ) ) AND NOT ( INDEXTERMS ( humans OR human ) ) )

*manually excluded the states that are no longer part of Europe

**Supplementary File 2:**

**Manual Search of Publicly Available Data on Seroprevalence of Tick-borne Infections: List of National and International Registries.**

International/European:

- ECDC (European Center of Disease Control): https://www.ecdc.europa.eu/en

National:

- Aland Islands: https://www.ahs.ax/
- Albania: https://shendetesia.gov.al/
- Andorra: https://www.salut.ad/
- Austria: https://www.ages.at/
- Belarus: https://minzdrav.gov.by/en/
- Belgium: https://www.health.belgium.be/en/about-fps
- Bosnia and Herzegovina
- Bulgaria: https://www.mh.government.bg/en/
- Channel Islands: https://www.gov.je/Health/
- Croatia: https://www.hzjz.hr/en/
- Czechia: https://mzd.gov.cz/en/the-ministry-of-health/
- Denmark: https://www.sst.dk/en/
- Estonia: https://www.terviseamet.ee/en
- Faeroe Islands: https://www.hmr.fo/en/
- Finland: https://thl.fi/
- France: https://www.santepubliquefrance.fr/
- Germany: https://www.rki.de/
- Gibraltar: https://www.gha.gi/public-health/
- Greece: https://eody.gov.gr/en/npho/
- Holy See: https://www.vaticanstate.va/it/direzioni/direzione-di-sanita-e-igiene.html
- Hungary: https://egeszsegvonal.gov.hu/en/
- Iceland: https://island.is/en/public-health
- Ireland: https://island.is/en/category/health
- Isle of Man: https://www.gov.im/publichealth
- Italy: https://www.iss.it/
- Kosovo: https://niph-rks.org/
- Latvia: https://www.vmnvd.gov.lv/en/health-care-latvia
- Liechtenstein: https://www.llv.li/en/national-administration/office-of-public-health/
- Lithuania: https://sam.lrv.lt/en/health-care/public-health/
- Luxembourg: https://m3s.gouvernement.lu/en.html
- Malta: https://health.gov.mt/
- Monaco: https://en.gouv.mc/Policy-Practice/Social-Affairs-and-Health/
- Montenegro: https://www.gov.me/en/government-of-montenegro/health
- Netherlands: https://www.government.nl/ministries/ministry-of-health-welfare-and-sport
- North Macedonia: https://iph.mk/en/
- Norway: https://www.fhi.no/en/
- Poland: https://welcome.uw.edu.pl/healthcare-in-poland/
- Portugal: https://www2.gov.pt/en/temas/saude
- Republic of Moldova: https://ms.gov.md/en/
- Romania: https://www.presidency.ro/en/presidential-administration/departments/department-for-public-health
- Russian Federation: http://government.ru/
- San Marino: https://www.gov.sm/
- Serbia: https://www.srbija.gov.rs/
- Slovakia: https://www.uvzsr.sk/web/uvzen
- Slovenia: https://nijz.si/en/
- Spain: https://www.isciii.es/servicios/vigilancia-salud-publica-renave
- Svalbard and Jan Mayen Islands
- Sweden: https://www.folkhalsomyndigheten.se/the-public-health-agency-of-sweden/
- Switzerland: https://www.bag.admin.ch/
- Ukraine: https://moz.gov.ua/
- United Kingdom of Great Britain and Northern Ireland: https://www.gov.uk/

**Supplementary File 3:**

**Risk of Bias Assessment of Included Studies using JBI Critical Appraisal Checklist for Studies Reporting Prevalence Data: Summary and Full Checklists.**

| **Nr.** | **Authors** | **Title** | **Appraisal** | **Reference** |
| --- | --- | --- | --- | --- |
| 1 | Ackermann-Gäumann et al. | Comparison of four commercial IgG-enzyme-linked immunosorbent assays for the detection of *Tick-Borne Encephalitis Virus* antibodies | Include | ^30^ |
| 2 | Ackermann-Gäumann et al. | Prevalence of anti-Tick-Borne Encephalitis *Virus* (*TBEV*) antibodies in Swiss blood donors in 2014-2015 | Include | ^31^ |
| 3 | Albinsson et al. | Seroprevalence of *Tick-Borne Encephalitis Virus* and vaccination coverage of Tick-Borne Encephalitis, Sweden, 2018 to 2019 | Include | ^32^ |
| 4 | Banović et al. | Shared odds of *Borrelia* and *Rabies Virus* exposure in Serbia | Include | ^33^ |
| 5 | Banović et al. | *Tick-Borne Encephalitis Virus* seropositivity among tick infested individuals in Serbia | Include | ^34^ |
| 6 | Barreiro-Hurlé et al. | Seroprevalence of Lyme disease in southwest Asturias | Include | ^35^ |
| 7 | Bazovska et al. | Reported incidence of Lyme disease in Slovakia and antibodies to *B. burgdorferi* antigens detected in healthy population | Include | ^36^ |
| 8 | Bloch et al. | Molecular Screening of Blood Donors for *Babesia* in Tyrol, Austria | Include | ^37^ |
| 9 | Borawski et al. | Assessment of *Coxiella burnetii* presence after tick bite in north-eastern Poland | Include | ^38^ |
| 10 | Borawski et al. | Prevalence of *Spotted Fever Group Rickettsia* in North-Eastern Poland | Include | ^39^ |
| 11 | Brouqui et al. | Ectoparasitism and vector-borne diseases in 930 homeless people from Marseilles | Include | ^40^ |
| 12 | Busson et al. | Evaluation of commercial screening tests and blot assays for the diagnosis of Lyme borreliosis | Include | ^41^ |
| 13 | Carlsson et al. | Subclinical Lyme borreliosis is common in south-eastern Sweden and may be distinguished from Lyme neuroborreliosis by sex, age and specific immune marker patterns | Include | ^42^ |
| 14 | Chmielewska-Badora et al. | Serological survey in persons occupationally exposed to tick-borne pathogens in cases of co-infections with *Borrelia burgdorferi, Anaplasma phagocytophilum, Bartonella spp. and Babesia microti* | Include | ^43^ |
| 15 | Chmielewski et al. | Tick-borne pathogens *Bartonella spp., Borrelia burgdorferi sensu lato, Coxiella burnetii* and *Rickettsia spp.* may trigger endocarditis | Include | ^44^ |
| 16 | Chmielewski et al. | Presence of *Bartonella spp*. in Various Human Populations | Include | ^45^ |
| 17 | Chochlakis et al. | A serosurvey of *Anaplasma phagocytophilum* in blood donors in Crete, Greece | Include | ^46^ |
| 18 | Cisak et al. | Risk of tick-borne bacterial diseases among workers of Roztocze National Park (south-eastern Poland) | Include | ^47^ |
| 19 | Coroian et al. | Seroprevalence Rates against *West Nile, Usutu,* and *Tick-Borne Encephalitis Viruses* in Blood-Donors from North-Western Romania | Include | ^48^ |
| 20 | De Keukeleire et al. | Seroprevalence of *Borrelia burgdorferi, Anaplasma phagocytophilum,* and *Francisella tularensis* Infections in Belgium: Results of Three Population-Based Samples | Include | ^49^ |
| 21 | Di Renzi et al. | Risk of acquiring tick-borne infections in forestry workers from Lazio, Italy | Include | ^50^ |
| 22 | Elfving et al. | Seroprevalence of *Rickettsia spp*. infection among tick-bitten patients and blood donors in Sweden | Include | ^51^ |
| 23 | Euringer et al. | *Tick-Borne Encephalitis Virus* IgG antibody surveillance: vaccination- and infection-induced seroprevalences, south-western Germany, 2021 | Include | ^52^ |
| 24 | Grzeszczuk et al. | Human anaplasmosis in north-eastern Poland: Seroprevalence in humans and prevalence in Ixodes ricinus ticks | Include | ^53^ |
| 25 | Gynthersen et al. | *Neoehrlichia mikurensis* is uncommon in rheumatological patients receiving tumour necrosis factor inhibitors and in blood donors: a retrospective cohort study | Include | ^54^ |
| 26 | Gynthersen et al. | *Neoehrlichia mikurensis* in Danish immunocompromised patients: a retrospective cohort study | Include | ^55^ |
| 27 | Hildebrandt et al. | First confirmed autochthonous case of human *Babesia microti* infection in Europe | Include | ^26^ |
| 28 | Hjetland et al. | Seroprevalence of antibodies to *Borrelia burgdorferi sensu lato* in healthy adults from western Norway: Risk factors and methodological aspects | Include | ^56^ |
| 29 | Hjetland et al. | Seroprevalence of antibodies to *Tick-Borne Encephalitis Virus* and A*naplasma phagocytophilum* in healthy adults from western Norway | Include | ^57^ |
| 30 | Hvidsten et al. | Blood donor *Borrelia burgdorferi sensu lato* seroprevalence and history of tick bites at a northern limit of the vector distribution | Include | ^58^ |
| 31 | Jensen et al. | Evaluation of factors influencing tick bites and tick-borne infections: a longitudinal study | Include | ^59^ |
| 32 | Jensen et al. | Rickettsiosis in Denmark: A nation-wide survey | Include | ^60^ |
| 33 | Johansson et al. | Significant variations in the seroprevalence of C6 ELISA antibodies in a highly endemic area for Lyme borreliosis: evaluation of age, sex and seasonal differences | Include | ^61^ |
| 34 | Jovanovic et al. | Seroprevalence of *Borrelia burgdorferi* in occupationally exposed persons in the Belgrade area, Serbia | Include | ^62^ |
| 35 | Kaczmarek et al. | Asymptomatic carrier of *Babesia spp.* among blood donors - epidemiological situation in Poland | Include | ^63^ |
| 36 | Kalmár et al. | Seroprevalence of antibodies against *Borrelia burgdorferi sensu lato* in healthy blood donors in Romania: an update | Include | ^64^ |
| 37 | Koetsveld et al. | Serological and molecular evidence for *Spotted Fever Group Rickettsia* and *Borrelia burgdorferi sensu lato* co-infections in The Netherlands | Include | ^65^ |
| 38 | Labbé Sandelin et al. | Detection of *Neoehrlichia mikurensis* DNA in blood donors in southeastern Sweden | Include | ^66^ |
| 39 | Larsen et al. | Detection of specific IgG antibodies in blood donors and *Tick-Borne Encephalitis Virus* in ticks within a non-endemic area in southeast Norway | Include | ^67^ |
| 40 | Lindblom et al. | Seroreactivity for *Spotted Fever Rickettsiae* and co-infections with other tick-borne agents among habitants in central and southern Sweden | Include | ^68^ |
| 41 | Łysakowska et al. | The seroprevalence of *Bartonella spp.* in the blood of patients with musculoskeletal complaints and blood donors, Poland: a pilot study | Include | ^69^ |
| 42 | Magyar et al. | New geographical area on the map of *Crimean-Congo Hemorrhagic Fever Virus*: First serological evidence in the Hungarian population | Include | ^70^ |
| 43 | Marvik et al. | Low prevalence of *Tick-Borne Encephalitis Virus* antibodies in Norwegian blood donors | Include | ^71^ |
| 44 | Monsalve Arteaga et al. | *Crimean-Congo haemorrhagic fever (CCHF) virus*-specific antibody detection in blood donors, Castile-León, Spain, summer 2017 and 2018 | Include | ^72^ |
| 45 | Müller et al. | Detection of *Bartonella* spp*.* in Ixodes ricinus ticks and *Bartonella* seroprevalence in human populations | Include | ^73^ |
| 46 | Munro et al. | Seroprevalence of Lyme borreliosis in Scottish blood donors | Include | ^74^ |
| 47 | Mygland et al. | Chronic polyneuropathy and Lyme disease | Include | ^75^ |
| 48 | Ocias et al. | Evidence of *Rickettsiae* in Danish patients tested for Lyme neuroborreliosis: a retrospective study of archival samples | Include | ^76^ |
| 49 | Pawełczyk et al. | Seroprevalence of six pathogens transmitted by the Ixodes ricinus ticks in asymptomatic individuals with HIV infection and in blood donors | Include | ^77^ |
| 50 | Santos et al. | Human exposure to *Anaplasma phagocytophilum* in Portugal | Include | ^78^ |
| 51 | Sonnleitner et al. | *Spotted Fever Group-Rickettsiae* in the Tyrols: evidence by seroepidemiology and PCR | Include | ^79^ |
| 52 | Sonnleitner et al. | Human seroprevalence against *Borrelia burgdorferi sensu lato* in two comparable regions of the eastern Alps is not correlated to vector infection rates | Include | ^80^ |
| 53 | Sonnleitner et al. | Risk assessment of transfusion-associated Babesiosis in Tyrol: appraisal by seroepidemiology and polymerase chain reaction | Include | ^81^ |
| 54 | Stańczak et al. | Kampinos National Park: a risk area for *Spotted Fever Group Rickettsioses*, central Poland? | Include | ^82^ |
| 55 | Tomasiewicz et al. | The risk of exposure to *Anaplasma phagocytophilum* infection in mid-eastern Poland | Include | ^83^ |
| 56 | Walder et al. | Serological evidence for Human Granulocytic Ehrlichiosis in Western Austria | Include | ^84^ |
| 57 | Zákutná et al. | Pilot Cross-Sectional Study of Three Zoonoses (Lyme Disease, Tularaemia, Leptospirosis) among Healthy Blood Donors in Eastern Slovakia | Include | ^85^ |

Summary: Risk of Bias Assessment of Included Studies using JBI Critical Appraisal Checklist for Studies Reporting Prevalence Data.

JBI Critical Appraisal Checklist for
studies reporting prevalence data

Reviewer Sophie Mathys Date 26.06.2024

Author Ackermann-Gäumann et al. Year 2019 Record Number_________

|  | Yes | No | Unclear | Not applicable |
| --- | --- | --- | --- | --- |
| 1. Was the sample frame appropriate to address the target population? | x | □ | □ | □ |
| 1. Were study participants sampled in an appropriate way? | x | □ | □ | □ |
| 1. Was the sample size adequate? | x | □ | □ | □ |
| 1. Were the study subjects and the setting described in detail? | □ | □ | x | □ |
| 1. Was the data analysis conducted with sufficient coverage of the identified sample? | □ | □ | x | □ |
| 1. Were valid methods used for the identification of the condition? | x | □ | □ | □ |
| 1. Was the condition measured in a standard, reliable way for all participants? | □ | □ | x | □ |
| 1. Was there appropriate statistical analysis? | □ | □ | x | □ |
| 1. Was the response rate adequate, and if not, was the low response rate managed appropriately? | □ | □ | □ | x |

Overall appraisal: Include x Exclude □ Seek further info □

Comments (Including reason for exclusion)

______________________________________________________________________________________________________________________________________________________________________________________

JBI Critical Appraisal Checklist for
studies reporting prevalence data

Reviewer Sophie Mathys Date 26.06.2024

Author__ Ackermann-Gäumann et al ___ Year 2023 Record Number_________

|  | Yes | No | Unclear | Not applicable |
| --- | --- | --- | --- | --- |
| 1. Was the sample frame appropriate to address the target population? | x | □ | □ | □ |
| 1. Were study participants sampled in an appropriate way? | □ | □ | x | □ |
| 1. Was the sample size adequate? | x | □ | □ | □ |
| 1. Were the study subjects and the setting described in detail? | □ | □ | x | □ |
| 1. Was the data analysis conducted with sufficient coverage of the identified sample? | □ | □ | x | □ |
| 1. Were valid methods used for the identification of the condition? | x | □ | □ | □ |
| 1. Was the condition measured in a standard, reliable way for all participants? | □ | □ | x | □ |
| 1. Was there appropriate statistical analysis? | □ | □ | x | □ |
| 1. Was the response rate adequate, and if not, was the low response rate managed appropriately? | □ | □ | □ | x |

Overall appraisal: Include x Exclude □ Seek further info □

Comments (Including reason for exclusion)

______________________________________________________________________________________________________________________________________________________________________________________

JBI Critical Appraisal Checklist for
studies reporting prevalence data

Reviewer Sophie Mathys Date 26.06.2024

Author___ Albinsson et al._______ Year 2024 Record Number_________

|  | Yes | No | Unclear | Not applicable |
| --- | --- | --- | --- | --- |
| 1. Was the sample frame appropriate to address the target population? | x | □ | □ | □ |
| 1. Were study participants sampled in an appropriate way? | x | □ | □ | □ |
| 1. Was the sample size adequate? | x | □ | □ | □ |
| 1. Were the study subjects and the setting described in detail? | □ | □ | x | □ |
| 1. Was the data analysis conducted with sufficient coverage of the identified sample? | □ | □ | x | □ |
| 1. Were valid methods used for the identification of the condition? | x | □ | □ | □ |
| 1. Was the condition measured in a standard, reliable way for all participants? | □ | □ | x | □ |
| 1. Was there appropriate statistical analysis? | □ | □ | x | □ |
| 1. Was the response rate adequate, and if not, was the low response rate managed appropriately? | □ | □ | □ | x |

Overall appraisal: Include x Exclude □ Seek further info □

Comments (Including reason for exclusion)

______________________________________________________________________________________________________________________________________________________________________________________

JBI Critical Appraisal Checklist for
studies reporting prevalence data

Reviewer Sophie Mathys Date 26.06.2024

Author___ Banović et al.______ Year 2021 Record Number_________

|  | Yes | No | Unclear | Not applicable |
| --- | --- | --- | --- | --- |
| 1. Was the sample frame appropriate to address the target population? | x | □ | □ | □ |
| 1. Were study participants sampled in an appropriate way? | □ | □ | x | □ |
| 1. Was the sample size adequate? | □ | □ | x | □ |
| 1. Were the study subjects and the setting described in detail? | x | □ | □ | □ |
| 1. Was the data analysis conducted with sufficient coverage of the identified sample? | □ | □ | x | □ |
| 1. Were valid methods used for the identification of the condition? | x | □ | □ | □ |
| 1. Was the condition measured in a standard, reliable way for all participants? | □ | □ | x | □ |
| 1. Was there appropriate statistical analysis? | □ | □ | x | □ |
| 1. Was the response rate adequate, and if not, was the low response rate managed appropriately? | □ | □ | □ | x |

Overall appraisal: Include x Exclude □ Seek further info □

Comments (Including reason for exclusion)

______________________________________________________________________________________________________________________________________________________________________________________

JBI Critical Appraisal Checklist for
studies reporting prevalence data

Reviewer Sophie Mathys Date 26.06.2024

Author_ Banović et al. ______ Year 2021 Record Number_________

|  | Yes | No | Unclear | Not applicable |
| --- | --- | --- | --- | --- |
| 1. Was the sample frame appropriate to address the target population? | x | □ | □ | □ |
| 1. Were study participants sampled in an appropriate way? | x | □ | □ | □ |
| 1. Was the sample size adequate? | □ | □ | x | □ |
| 1. Were the study subjects and the setting described in detail? | x | □ | □ | □ |
| 1. Was the data analysis conducted with sufficient coverage of the identified sample? | □ | □ | x | □ |
| 1. Were valid methods used for the identification of the condition? | x | □ | □ | □ |
| 1. Was the condition measured in a standard, reliable way for all participants? | x | □ | □ | □ |
| 1. Was there appropriate statistical analysis? | □ | □ | x | □ |
| 1. Was the response rate adequate, and if not, was the low response rate managed appropriately? | □ | □ | □ | x |

Overall appraisal: Include x Exclude □ Seek further info □

Comments (Including reason for exclusion)

______________________________________________________________________________________________________________________________________________________________________________________

JBI Critical Appraisal Checklist for
studies reporting prevalence data

Reviewer Sophie Mathys Date 26.06.2024

Author____Barreiro-Hurlé et al_____ Year 2020 Record Number_________

|  | Yes | No | Unclear | Not applicable |
| --- | --- | --- | --- | --- |
| 1. Was the sample frame appropriate to address the target population? | x | □ | □ | □ |
| 1. Were study participants sampled in an appropriate way? | □ | □ | x | □ |
| 1. Was the sample size adequate? | □ | □ | x | □ |
| 1. Were the study subjects and the setting described in detail? | x | □ | □ | □ |
| 1. Was the data analysis conducted with sufficient coverage of the identified sample? | □ | □ | x | □ |
| 1. Were valid methods used for the identification of the condition? | x | □ | □ | □ |
| 1. Was the condition measured in a standard, reliable way for all participants? | □ | □ | x | □ |
| 1. Was there appropriate statistical analysis? | □ | □ | x | □ |
| 1. Was the response rate adequate, and if not, was the low response rate managed appropriately? | □ | □ | □ | x |

Overall appraisal: Include x Exclude □ Seek further info □

Comments (Including reason for exclusion)

______________________________________________________________________________________________________________________________________________________________________________________

JBI Critical Appraisal Checklist for
studies reporting prevalence data

Reviewer Sophie Mathys Date 26.06.2024

Author____ Bazovska et al_____ Year 2005 Record Number_________

|  | Yes | No | Unclear | Not applicable |
| --- | --- | --- | --- | --- |
| 1. Was the sample frame appropriate to address the target population? | x | □ | □ | □ |
| 1. Were study participants sampled in an appropriate way? | □ | □ | x | □ |
| 1. Was the sample size adequate? | x | □ | □ | □ |
| 1. Were the study subjects and the setting described in detail? | □ | x | □ | □ |
| 1. Was the data analysis conducted with sufficient coverage of the identified sample? | □ | □ | x | □ |
| 1. Were valid methods used for the identification of the condition? | x | □ | □ | □ |
| 1. Was the condition measured in a standard, reliable way for all participants? | □ | □ | x | □ |
| 1. Was there appropriate statistical analysis? | □ | □ | x | □ |
| 1. Was the response rate adequate, and if not, was the low response rate managed appropriately? | □ | □ | □ | x |

Overall appraisal: Include x Exclude □ Seek further info □

Comments (Including reason for exclusion)

______________________________________________________________________________________________________________________________________________________________________________________

JBI Critical Appraisal Checklist for
studies reporting prevalence data

Reviewer Sophie Mathys Date 26.06.2024

Author___Bloch et al_____ Year 2023 Record Number_________

|  | Yes | No | Unclear | Not applicable |
| --- | --- | --- | --- | --- |
| 1. Was the sample frame appropriate to address the target population? | x | □ | □ | □ |
| 1. Were study participants sampled in an appropriate way? | □ | □ | x | □ |
| 1. Was the sample size adequate? | x | □ | □ | □ |
| 1. Were the study subjects and the setting described in detail? | x | □ | □ | □ |
| 1. Was the data analysis conducted with sufficient coverage of the identified sample? | □ | □ | x | □ |
| 1. Were valid methods used for the identification of the condition? | x | □ | □ | □ |
| 1. Was the condition measured in a standard, reliable way for all participants? | □ | □ | x | □ |
| 1. Was there appropriate statistical analysis? | □ | □ | x | □ |
| 1. Was the response rate adequate, and if not, was the low response rate managed appropriately? | □ | □ | □ | x |

Overall appraisal: Include x Exclude □ Seek further info □

Comments (Including reason for exclusion)

______________________________________________________________________________________________________________________________________________________________________________________

JBI Critical Appraisal Checklist for
studies reporting prevalence data

Reviewer Sophie Mathys Date 26.06.2024

Author____Borawski et al___ Year 2020 Record Number_________

|  | Yes | No | Unclear | Not applicable |
| --- | --- | --- | --- | --- |
| 1. Was the sample frame appropriate to address the target population? | x | □ | □ | □ |
| 1. Were study participants sampled in an appropriate way? | x | □ | □ | □ |
| 1. Was the sample size adequate? | □ | x | □ | □ |
| 1. Were the study subjects and the setting described in detail? | x | □ | □ | □ |
| 1. Was the data analysis conducted with sufficient coverage of the identified sample? | □ | □ | x | □ |
| 1. Were valid methods used for the identification of the condition? | x | □ | □ | □ |
| 1. Was the condition measured in a standard, reliable way for all participants? | x | □ | □ | □ |
| 1. Was there appropriate statistical analysis? | □ | □ | x | □ |
| 1. Was the response rate adequate, and if not, was the low response rate managed appropriately? | □ | □ | □ | x |

Overall appraisal: Include x Exclude □ Seek further info □

Comments (Including reason for exclusion)

______________________________________________________________________________________________________________________________________________________________________________________

JBI Critical Appraisal Checklist for
studies reporting prevalence data

Reviewer Sophie Mathys Date 26.06.2024

Author___ Borawski et al___ _______ Year 2019 Record Number_________

|  | Yes | No | Unclear | Not applicable |
| --- | --- | --- | --- | --- |
| 1. Was the sample frame appropriate to address the target population? | x | □ | □ | □ |
| 1. Were study participants sampled in an appropriate way? | x | □ | □ | □ |
| 1. Was the sample size adequate? | □ | x | □ | □ |
| 1. Were the study subjects and the setting described in detail? | x | □ | □ | □ |
| 1. Was the data analysis conducted with sufficient coverage of the identified sample? | □ | □ | x | □ |
| 1. Were valid methods used for the identification of the condition? | x | □ | □ | □ |
| 1. Was the condition measured in a standard, reliable way for all participants? | □ | □ | x | □ |
| 1. Was there appropriate statistical analysis? | □ | □ | x | □ |
| 1. Was the response rate adequate, and if not, was the low response rate managed appropriately? | □ | □ | □ | x |

Overall appraisal: Include x Exclude □ Seek further info □

Comments (Including reason for exclusion)

______________________________________________________________________________________________________________________________________________________________________________________

JBI Critical Appraisal Checklist for
studies reporting prevalence data

Reviewer Sophie Mathys Date 26.06.2024

Author __Brouqui et al_____ Year 2005 Record Number_________

|  | Yes | No | Unclear | Not applicable |
| --- | --- | --- | --- | --- |
| 1. Was the sample frame appropriate to address the target population? | x | □ | □ | □ |
| 1. Were study participants sampled in an appropriate way? | □ | □ | x | □ |
| 1. Was the sample size adequate? | □ | x | □ | □ |
| 1. Were the study subjects and the setting described in detail? | x | □ | □ | □ |
| 1. Was the data analysis conducted with sufficient coverage of the identified sample? | □ | □ | x | □ |
| 1. Were valid methods used for the identification of the condition? | x | □ | □ | □ |
| 1. Was the condition measured in a standard, reliable way for all participants? | □ | □ | x | □ |
| 1. Was there appropriate statistical analysis? | □ | □ | x | □ |
| 1. Was the response rate adequate, and if not, was the low response rate managed appropriately? | □ | □ | □ | x |

Overall appraisal: Include x Exclude □ Seek further info □

Comments (Including reason for exclusion)

______________________________________________________________________________________________________________________________________________________________________________________

JBI Critical Appraisal Checklist for
studies reporting prevalence data

Reviewer Sophie Mathys Date 26.06.2024

Author___Busson et al_____ Year 2012 Record Number_________

|  | Yes | No | Unclear | Not applicable |
| --- | --- | --- | --- | --- |
| 1. Was the sample frame appropriate to address the target population? | x | □ | □ | □ |
| 1. Were study participants sampled in an appropriate way? | x | □ | □ | □ |
| 1. Was the sample size adequate? | □ | x | □ | □ |
| 1. Were the study subjects and the setting described in detail? | x | □ | □ | □ |
| 1. Was the data analysis conducted with sufficient coverage of the identified sample? | □ | □ | x | □ |
| 1. Were valid methods used for the identification of the condition? | x | □ | □ | □ |
| 1. Was the condition measured in a standard, reliable way for all participants? | □ | □ | x | □ |
| 1. Was there appropriate statistical analysis? | □ | □ | x | □ |
| 1. Was the response rate adequate, and if not, was the low response rate managed appropriately? | □ | □ | □ | x |

Overall appraisal: Include x Exclude □ Seek further info □

Comments (Including reason for exclusion)

______________________________________________________________________________________________________________________________________________________________________________________

JBI Critical Appraisal Checklist for
studies reporting prevalence data

Reviewer Sophie Mathys Date 26.06.2024

Author____Carlsson et al. ____ Year 2018 Record Number_________

|  | Yes | No | Unclear | Not applicable |
| --- | --- | --- | --- | --- |
| 1. Was the sample frame appropriate to address the target population? | x | □ | □ | □ |
| 1. Were study participants sampled in an appropriate way? | x | □ | □ | □ |
| 1. Was the sample size adequate? | x | □ | □ | □ |
| 1. Were the study subjects and the setting described in detail? | x | □ | □ | □ |
| 1. Was the data analysis conducted with sufficient coverage of the identified sample? | □ | □ | x | □ |
| 1. Were valid methods used for the identification of the condition? | x | □ | □ | □ |
| 1. Was the condition measured in a standard, reliable way for all participants? | □ | □ | x | □ |
| 1. Was there appropriate statistical analysis? | □ | □ | x | □ |
| 1. Was the response rate adequate, and if not, was the low response rate managed appropriately? | □ | □ | □ | x |

Overall appraisal: Include x Exclude □ Seek further info □

Comments (Including reason for exclusion)

______________________________________________________________________________________________________________________________________________________________________________________

JBI Critical Appraisal Checklist for
studies reporting prevalence data

Reviewer Sophie Mathys Date 26.06.2024

Author Chmielewska-Badora et al. Year 2012 Record Number_________

|  | Yes | No | Unclear | Not applicable |
| --- | --- | --- | --- | --- |
| 1. Was the sample frame appropriate to address the target population? | x | □ | □ | □ |
| 1. Were study participants sampled in an appropriate way? | x | □ | □ | □ |
| 1. Was the sample size adequate? | □ | x | □ | □ |
| 1. Were the study subjects and the setting described in detail? | □ | x | □ | □ |
| 1. Was the data analysis conducted with sufficient coverage of the identified sample? | □ | □ | x | □ |
| 1. Were valid methods used for the identification of the condition? | x | □ | □ | □ |
| 1. Was the condition measured in a standard, reliable way for all participants? | □ | □ | x | □ |
| 1. Was there appropriate statistical analysis? | □ | □ | x | □ |
| 1. Was the response rate adequate, and if not, was the low response rate managed appropriately? | □ | □ | □ | x |

Overall appraisal: Include x Exclude □ Seek further info □

Comments (Including reason for exclusion)

______________________________________________________________________________________________________________________________________________________________________________________

JBI Critical Appraisal Checklist for
studies reporting prevalence data

Reviewer Sophie Mathys Date 26.06.2024

Author____ Chmielewski et al.__ Year 2019 Record Number_________

|  | Yes | No | Unclear | Not applicable |
| --- | --- | --- | --- | --- |
| 1. Was the sample frame appropriate to address the target population? | x | □ | □ | □ |
| 1. Were study participants sampled in an appropriate way? | □ | □ | x | □ |
| 1. Was the sample size adequate? | □ | x | □ | □ |
| 1. Were the study subjects and the setting described in detail? | x | □ | □ | □ |
| 1. Was the data analysis conducted with sufficient coverage of the identified sample? | □ | □ | x | □ |
| 1. Were valid methods used for the identification of the condition? | x | □ | □ | □ |
| 1. Was the condition measured in a standard, reliable way for all participants? | □ | □ | x | □ |
| 1. Was there appropriate statistical analysis? | □ | □ | x | □ |
| 1. Was the response rate adequate, and if not, was the low response rate managed appropriately? | □ | □ | □ | x |

Overall appraisal: Include x Exclude □ Seek further info □

Comments (Including reason for exclusion)

______________________________________________________________________________________________________________________________________________________________________________________

JBI Critical Appraisal Checklist for
studies reporting prevalence data

Reviewer Sophie Mathys Date 26.06.2024

Author Chmielewski et al. Year 2007 Record Number_________

|  | Yes | No | Unclear | Not applicable |
| --- | --- | --- | --- | --- |
| 1. Was the sample frame appropriate to address the target population? | x | □ | □ | □ |
| 1. Were study participants sampled in an appropriate way? | □ | □ | x | □ |
| 1. Was the sample size adequate? | x | □ | □ | □ |
| 1. Were the study subjects and the setting described in detail? | x | □ | □ | □ |
| 1. Was the data analysis conducted with sufficient coverage of the identified sample? | □ | □ | x | □ |
| 1. Were valid methods used for the identification of the condition? | x | □ | □ | □ |
| 1. Was the condition measured in a standard, reliable way for all participants? | □ | □ | x | □ |
| 1. Was there appropriate statistical analysis? | □ | □ | x | □ |
| 1. Was the response rate adequate, and if not, was the low response rate managed appropriately? | □ | □ | □ | x |

Overall appraisal: Include x Exclude □ Seek further info □

Comments (Including reason for exclusion)

______________________________________________________________________________________________________________________________________________________________________________________

JBI Critical Appraisal Checklist for
studies reporting prevalence data

Reviewer Sophie Mathys Date 26.06.2024

Author Chochlakis et al. Year 2008 Record Number_________

|  | Yes | No | Unclear | Not applicable |
| --- | --- | --- | --- | --- |
| 1. Was the sample frame appropriate to address the target population? | x | □ | □ | □ |
| 1. Were study participants sampled in an appropriate way? | x | □ | □ | □ |
| 1. Was the sample size adequate? | x | □ | □ | □ |
| 1. Were the study subjects and the setting described in detail? | x | □ | □ | □ |
| 1. Was the data analysis conducted with sufficient coverage of the identified sample? | □ | □ | x | □ |
| 1. Were valid methods used for the identification of the condition? | x | □ | □ | □ |
| 1. Was the condition measured in a standard, reliable way for all participants? | □ | □ | x | □ |
| 1. Was there appropriate statistical analysis? | □ | □ | x | □ |
| 1. Was the response rate adequate, and if not, was the low response rate managed appropriately? | □ | □ | □ | x |

Overall appraisal: Include x Exclude □ Seek further info □

Comments (Including reason for exclusion)

______________________________________________________________________________________________________________________________________________________________________________________

JBI Critical Appraisal Checklist for
studies reporting prevalence data

Reviewer Sophie Mathys Date 26.06.2024

Author Cisak et al. Year 2005 Record Number_________

|  | Yes | No | Unclear | Not applicable |
| --- | --- | --- | --- | --- |
| 1. Was the sample frame appropriate to address the target population? | x | □ | □ | □ |
| 1. Were study participants sampled in an appropriate way? | □ | □ | x | □ |
| 1. Was the sample size adequate? | □ | x | □ | □ |
| 1. Were the study subjects and the setting described in detail? | x | □ | □ | □ |
| 1. Was the data analysis conducted with sufficient coverage of the identified sample? | □ | □ | x | □ |
| 1. Were valid methods used for the identification of the condition? | x | □ | □ | □ |
| 1. Was the condition measured in a standard, reliable way for all participants? | □ | □ | x | □ |
| 1. Was there appropriate statistical analysis? | □ | □ | x | □ |
| 1. Was the response rate adequate, and if not, was the low response rate managed appropriately? | □ | □ | □ | x |

Overall appraisal: Include x Exclude □ Seek further info □

Comments (Including reason for exclusion)

______________________________________________________________________________________________________________________________________________________________________________________

JBI Critical Appraisal Checklist for
studies reporting prevalence data

Reviewer Sophie Mathys Date 26.06.2024

Author Coroian et al. Year 2022 Record Number_________

|  | Yes | No | Unclear | Not applicable |
| --- | --- | --- | --- | --- |
| 1. Was the sample frame appropriate to address the target population? | x | □ | □ | □ |
| 1. Were study participants sampled in an appropriate way? | x | □ | □ | □ |
| 1. Was the sample size adequate? | x | □ | □ | □ |
| 1. Were the study subjects and the setting described in detail? | x | □ | □ | □ |
| 1. Was the data analysis conducted with sufficient coverage of the identified sample? | □ | □ | x | □ |
| 1. Were valid methods used for the identification of the condition? | x | □ | □ | □ |
| 1. Was the condition measured in a standard, reliable way for all participants? | x | □ | □ | □ |
| 1. Was there appropriate statistical analysis? | □ | □ | x | □ |
| 1. Was the response rate adequate, and if not, was the low response rate managed appropriately? | □ | □ | □ | x |

Overall appraisal: Include x Exclude □ Seek further info □

Comments (Including reason for exclusion)

______________________________________________________________________________________________________________________________________________________________________________________

JBI Critical Appraisal Checklist for
studies reporting prevalence data

Reviewer Sophie Mathys Date 26.06.2024

Author De Keukeleire et al. Year 2017 Record Number_________

|  | Yes | No | Unclear | Not applicable |
| --- | --- | --- | --- | --- |
| 1. Was the sample frame appropriate to address the target population? | x | □ | □ | □ |
| 1. Were study participants sampled in an appropriate way? | x | □ | □ | □ |
| 1. Was the sample size adequate? | x | □ | □ | □ |
| 1. Were the study subjects and the setting described in detail? | x | □ | □ | □ |
| 1. Was the data analysis conducted with sufficient coverage of the identified sample? | □ | □ | x | □ |
| 1. Were valid methods used for the identification of the condition? | x | □ | □ | □ |
| 1. Was the condition measured in a standard, reliable way for all participants? | □ | □ | x | □ |
| 1. Was there appropriate statistical analysis? | □ | □ | x | □ |
| 1. Was the response rate adequate, and if not, was the low response rate managed appropriately? | □ | □ | □ | x |

Overall appraisal: Include x Exclude □ Seek further info □

Comments (Including reason for exclusion)

______________________________________________________________________________________________________________________________________________________________________________________

JBI Critical Appraisal Checklist for
studies reporting prevalence data

Reviewer Sophie Mathys Date 26.06.2024

Author Di Renzi et al. Year 2010 Record Number_________

|  | Yes | No | Unclear | Not applicable |
| --- | --- | --- | --- | --- |
| 1. Was the sample frame appropriate to address the target population? | x | □ | □ | □ |
| 1. Were study participants sampled in an appropriate way? | □ | □ | x | □ |
| 1. Was the sample size adequate? | x | □ | □ | □ |
| 1. Were the study subjects and the setting described in detail? | x | □ | □ | □ |
| 1. Was the data analysis conducted with sufficient coverage of the identified sample? | □ | □ | x | □ |
| 1. Were valid methods used for the identification of the condition? | x | □ | □ | □ |
| 1. Was the condition measured in a standard, reliable way for all participants? | □ | □ | x | □ |
| 1. Was there appropriate statistical analysis? | □ | □ | x | □ |
| 1. Was the response rate adequate, and if not, was the low response rate managed appropriately? | □ | □ | □ | x |

Overall appraisal: Include x Exclude □ Seek further info □

Comments (Including reason for exclusion)

______________________________________________________________________________________________________________________________________________________________________________________

JBI Critical Appraisal Checklist for
studies reporting prevalence data

Reviewer Sophie Mathys Date 26.06.2024

Author Dobler et al. Year 2023 Record Number_________

|  | Yes | No | Unclear | Not applicable |
| --- | --- | --- | --- | --- |
| 1. Was the sample frame appropriate to address the target population? | x | □ | □ | □ |
| 1. Were study participants sampled in an appropriate way? | □ | □ | x | □ |
| 1. Was the sample size adequate? | x | □ | □ | □ |
| 1. Were the study subjects and the setting described in detail? | □ | □ | x | □ |
| 1. Was the data analysis conducted with sufficient coverage of the identified sample? | □ | □ | x | □ |
| 1. Were valid methods used for the identification of the condition? | x | □ | □ | □ |
| 1. Was the condition measured in a standard, reliable way for all participants? | □ | □ | x | □ |
| 1. Was there appropriate statistical analysis? | □ | □ | x | □ |
| 1. Was the response rate adequate, and if not, was the low response rate managed appropriately? | □ | □ | □ | x |

Overall appraisal: Include x Exclude □ Seek further info □

Comments (Including reason for exclusion)

______________________________________________________________________________________________________________________________________________________________________________________

JBI Critical Appraisal Checklist for
studies reporting prevalence data

Reviewer Sophie Mathys Date 26.06.2024

Author Elfving et al. Year 2008 Record Number_________

|  | Yes | No | Unclear | Not applicable |
| --- | --- | --- | --- | --- |
| 1. Was the sample frame appropriate to address the target population? | x | □ | □ | □ |
| 1. Were study participants sampled in an appropriate way? | □ | □ | x | □ |
| 1. Was the sample size adequate? | □ | x | □ | □ |
| 1. Were the study subjects and the setting described in detail? | □ | □ | x | □ |
| 1. Was the data analysis conducted with sufficient coverage of the identified sample? | □ | □ | x | □ |
| 1. Were valid methods used for the identification of the condition? | x | □ | □ | □ |
| 1. Was the condition measured in a standard, reliable way for all participants? | □ | □ | x | □ |
| 1. Was there appropriate statistical analysis? | □ | □ | x | □ |
| 1. Was the response rate adequate, and if not, was the low response rate managed appropriately? | □ | □ | □ | x |

Overall appraisal: Include x Exclude □ Seek further info □

Comments (Including reason for exclusion)

______________________________________________________________________________________________________________________________________________________________________________________

JBI Critical Appraisal Checklist for
studies reporting prevalence data

Reviewer Sophie Mathys Date 26.06.2024

Author Euringer et al. Year 2023 Record Number_________

|  | Yes | No | Unclear | Not applicable |
| --- | --- | --- | --- | --- |
| 1. Was the sample frame appropriate to address the target population? | x | □ | □ | □ |
| 1. Were study participants sampled in an appropriate way? | x | □ | □ | □ |
| 1. Was the sample size adequate? | □ | x | □ | □ |
| 1. Were the study subjects and the setting described in detail? | x | □ | □ | □ |
| 1. Was the data analysis conducted with sufficient coverage of the identified sample? | □ | □ | x | □ |
| 1. Were valid methods used for the identification of the condition? | x | □ | □ | □ |
| 1. Was the condition measured in a standard, reliable way for all participants? | □ | □ | x | □ |
| 1. Was there appropriate statistical analysis? | □ | □ | x | □ |
| 1. Was the response rate adequate, and if not, was the low response rate managed appropriately? | □ | □ | □ | x |

Overall appraisal: Include x Exclude □ Seek further info □

Comments (Including reason for exclusion)

______________________________________________________________________________________________________________________________________________________________________________________

JBI Critical Appraisal Checklist for
studies reporting prevalence data

Reviewer Sophie Mathys Date 26.06.2024

Author Grzeszczk et al. Year 2004 Record Number_________

|  | Yes | No | Unclear | Not applicable |
| --- | --- | --- | --- | --- |
| 1. Was the sample frame appropriate to address the target population? | x | □ | □ | □ |
| 1. Were study participants sampled in an appropriate way? | x | □ | □ | □ |
| 1. Was the sample size adequate? | □ | x | □ | □ |
| 1. Were the study subjects and the setting described in detail? | x | □ | □ | □ |
| 1. Was the data analysis conducted with sufficient coverage of the identified sample? | □ | □ | x | □ |
| 1. Were valid methods used for the identification of the condition? | x | □ | □ | □ |
| 1. Was the condition measured in a standard, reliable way for all participants? | □ | □ | x | □ |
| 1. Was there appropriate statistical analysis? | □ | □ | x | □ |
| 1. Was the response rate adequate, and if not, was the low response rate managed appropriately? | □ | □ | □ | x |

Overall appraisal: Include x Exclude □ Seek further info □

Comments (Including reason for exclusion)

______________________________________________________________________________________________________________________________________________________________________________________

JBI Critical Appraisal Checklist for
studies reporting prevalence data

Reviewer Sophie Mathys Date 26.06.2024

Author Gynthersen et al. Year 2024 Record Number_________

|  | Yes | No | Unclear | Not applicable |
| --- | --- | --- | --- | --- |
| 1. Was the sample frame appropriate to address the target population? | x | □ | □ | □ |
| 1. Were study participants sampled in an appropriate way? | x | □ | □ | □ |
| 1. Was the sample size adequate? | x | □ | □ | □ |
| 1. Were the study subjects and the setting described in detail? | x | □ | □ | □ |
| 1. Was the data analysis conducted with sufficient coverage of the identified sample? | □ | □ | x | □ |
| 1. Were valid methods used for the identification of the condition? | x | □ | □ | □ |
| 1. Was the condition measured in a standard, reliable way for all participants? | □ | □ | x | □ |
| 1. Was there appropriate statistical analysis? | □ | □ | x | □ |
| 1. Was the response rate adequate, and if not, was the low response rate managed appropriately? | □ | □ | □ | x |

Overall appraisal: Include x Exclude □ Seek further info □

Comments (Including reason for exclusion)

______________________________________________________________________________________________________________________________________________________________________________________

JBI Critical Appraisal Checklist for
studies reporting prevalence data

Reviewer Sophie Mathys Date 26.06.2024

Author Gynthersen et al. Year 2023 Record Number_________

|  | Yes | No | Unclear | Not applicable |
| --- | --- | --- | --- | --- |
| 1. Was the sample frame appropriate to address the target population? | x | □ | □ | □ |
| 1. Were study participants sampled in an appropriate way? | x | □ | □ | □ |
| 1. Was the sample size adequate? | □ | □ | x | □ |
| 1. Were the study subjects and the setting described in detail? | x | □ | □ | □ |
| 1. Was the data analysis conducted with sufficient coverage of the identified sample? | □ | □ | x | □ |
| 1. Were valid methods used for the identification of the condition? | x | □ | □ | □ |
| 1. Was the condition measured in a standard, reliable way for all participants? | □ | □ | x | □ |
| 1. Was there appropriate statistical analysis? | □ | □ | x | □ |
| 1. Was the response rate adequate, and if not, was the low response rate managed appropriately? | □ | □ | □ | x |

Overall appraisal: Include x Exclude □ Seek further info □

Comments (Including reason for exclusion)

______________________________________________________________________________________________________________________________________________________________________________________

JBI Critical Appraisal Checklist for
studies reporting prevalence data

Reviewer Sophie Mathys Date 26.06.2024

Author Hildebrandt et al. Year 2007 Record Number_________

|  | Yes | No | Unclear | Not applicable |
| --- | --- | --- | --- | --- |
| 1. Was the sample frame appropriate to address the target population? | x | □ | □ | □ |
| 1. Were study participants sampled in an appropriate way? | x | □ | □ | □ |
| 1. Was the sample size adequate? | □ | □ | x | □ |
| 1. Were the study subjects and the setting described in detail? | x | □ | □ | □ |
| 1. Was the data analysis conducted with sufficient coverage of the identified sample? | □ | □ | x | □ |
| 1. Were valid methods used for the identification of the condition? | x | □ | □ | □ |
| 1. Was the condition measured in a standard, reliable way for all participants? | □ | □ | x | □ |
| 1. Was there appropriate statistical analysis? | □ | □ | x | □ |
| 1. Was the response rate adequate, and if not, was the low response rate managed appropriately? | □ | □ | □ | x |

Overall appraisal: Include x Exclude □ Seek further info □

Comments (Including reason for exclusion)

______________________________________________________________________________________________________________________________________________________________________________________

JBI Critical Appraisal Checklist for
studies reporting prevalence data

Reviewer Sophie Mathys Date 26.06.2024

Author Hjetland et al. Year 2014 Record Number_________

|  | Yes | No | Unclear | Not applicable |
| --- | --- | --- | --- | --- |
| 1. Was the sample frame appropriate to address the target population? | x | □ | □ | □ |
| 1. Were study participants sampled in an appropriate way? | □ | □ | x | □ |
| 1. Was the sample size adequate? | x | □ | □ | □ |
| 1. Were the study subjects and the setting described in detail? | x | □ | □ | □ |
| 1. Was the data analysis conducted with sufficient coverage of the identified sample? | □ | □ | x | □ |
| 1. Were valid methods used for the identification of the condition? | x | □ | □ | □ |
| 1. Was the condition measured in a standard, reliable way for all participants? | □ | □ | x | □ |
| 1. Was there appropriate statistical analysis? | □ | □ | x | □ |
| 1. Was the response rate adequate, and if not, was the low response rate managed appropriately? | □ | □ | □ | x |

Overall appraisal: Include x Exclude □ Seek further info □

Comments (Including reason for exclusion)

______________________________________________________________________________________________________________________________________________________________________________________

JBI Critical Appraisal Checklist for
studies reporting prevalence data

Reviewer Sophie Mathys Date 26.06.2024

Author Hjetland et al. Year 2015 Record Number_________

|  | Yes | No | Unclear | Not applicable |
| --- | --- | --- | --- | --- |
| 1. Was the sample frame appropriate to address the target population? | x | □ | □ | □ |
| 1. Were study participants sampled in an appropriate way? | □ | □ | x | □ |
| 1. Was the sample size adequate? | x | □ | □ | □ |
| 1. Were the study subjects and the setting described in detail? | □ | □ | x | □ |
| 1. Was the data analysis conducted with sufficient coverage of the identified sample? | □ | □ | x | □ |
| 1. Were valid methods used for the identification of the condition? | x | □ | □ | □ |
| 1. Was the condition measured in a standard, reliable way for all participants? | □ | □ | x | □ |
| 1. Was there appropriate statistical analysis? | □ | □ | x | □ |
| 1. Was the response rate adequate, and if not, was the low response rate managed appropriately? | □ | □ | □ | x |

Overall appraisal: Include x Exclude □ Seek further info □

Comments (Including reason for exclusion)

______________________________________________________________________________________________________________________________________________________________________________________

JBI Critical Appraisal Checklist for
studies reporting prevalence data

Reviewer Sophie Mathys Date 26.06.2024

Author Hvidsten et al. Year 2017 Record Number_________

|  | Yes | No | Unclear | Not applicable |
| --- | --- | --- | --- | --- |
| 1. Was the sample frame appropriate to address the target population? | x | □ | □ | □ |
| 1. Were study participants sampled in an appropriate way? | □ | □ | x | □ |
| 1. Was the sample size adequate? | x | □ | □ | □ |
| 1. Were the study subjects and the setting described in detail? | x | □ | □ | □ |
| 1. Was the data analysis conducted with sufficient coverage of the identified sample? | □ | □ | x | □ |
| 1. Were valid methods used for the identification of the condition? | x | □ | □ | □ |
| 1. Was the condition measured in a standard, reliable way for all participants? | □ | □ | x | □ |
| 1. Was there appropriate statistical analysis? | □ | □ | x | □ |
| 1. Was the response rate adequate, and if not, was the low response rate managed appropriately? | □ | □ | □ | x |

Overall appraisal: Include x Exclude □ Seek further info □

Comments (Including reason for exclusion)

______________________________________________________________________________________________________________________________________________________________________________________

JBI Critical Appraisal Checklist for
studies reporting prevalence data

Reviewer Sophie Mathys Date 26.06.2024

Author Jensen et al. Year 2021 Record Number_________

|  | Yes | No | Unclear | Not applicable |
| --- | --- | --- | --- | --- |
| 1. Was the sample frame appropriate to address the target population? | x | □ | □ | □ |
| 1. Were study participants sampled in an appropriate way? | □ | □ | x | □ |
| 1. Was the sample size adequate? | x | □ | □ | □ |
| 1. Were the study subjects and the setting described in detail? | x | □ | □ | □ |
| 1. Was the data analysis conducted with sufficient coverage of the identified sample? | □ | □ | x | □ |
| 1. Were valid methods used for the identification of the condition? | x | □ | □ | □ |
| 1. Was the condition measured in a standard, reliable way for all participants? | □ | □ | x | □ |
| 1. Was there appropriate statistical analysis? | □ | □ | x | □ |
| 1. Was the response rate adequate, and if not, was the low response rate managed appropriately? | □ | □ | □ | x |

Overall appraisal: Include x Exclude □ Seek further info □

Comments (Including reason for exclusion)

______________________________________________________________________________________________________________________________________________________________________________________

JBI Critical Appraisal Checklist for
studies reporting prevalence data

Reviewer Sophie Mathys Date 26.06.2024

Author Jensen et al. Year 2023 Record Number_________

|  | Yes | No | Unclear | Not applicable |
| --- | --- | --- | --- | --- |
| 1. Was the sample frame appropriate to address the target population? | x | □ | □ | □ |
| 1. Were study participants sampled in an appropriate way? | □ | □ | x | □ |
| 1. Was the sample size adequate? | □ | x | □ | □ |
| 1. Were the study subjects and the setting described in detail? | x | □ | □ | □ |
| 1. Was the data analysis conducted with sufficient coverage of the identified sample? | □ | □ | x | □ |
| 1. Were valid methods used for the identification of the condition? | x | □ | □ | □ |
| 1. Was the condition measured in a standard, reliable way for all participants? | □ | □ | x | □ |
| 1. Was there appropriate statistical analysis? | □ | □ | x | □ |
| 1. Was the response rate adequate, and if not, was the low response rate managed appropriately? | □ | □ | □ | x |

Overall appraisal: Include x Exclude □ Seek further info □

Comments (Including reason for exclusion)

______________________________________________________________________________________________________________________________________________________________________________________

JBI Critical Appraisal Checklist for
studies reporting prevalence data

Reviewer Sophie Mathys Date 26.06.2024

Author Johansson et al. Year 2017 Record Number_________

|  | Yes | No | Unclear | Not applicable |
| --- | --- | --- | --- | --- |
| 1. Was the sample frame appropriate to address the target population? | x | □ | □ | □ |
| 1. Were study participants sampled in an appropriate way? | □ | □ | x | □ |
| 1. Was the sample size adequate? | x | □ | □ | □ |
| 1. Were the study subjects and the setting described in detail? | x | □ | □ | □ |
| 1. Was the data analysis conducted with sufficient coverage of the identified sample? | □ | □ | x | □ |
| 1. Were valid methods used for the identification of the condition? | x | □ | □ | □ |
| 1. Was the condition measured in a standard, reliable way for all participants? | □ | □ | x | □ |
| 1. Was there appropriate statistical analysis? | □ | □ | x | □ |
| 1. Was the response rate adequate, and if not, was the low response rate managed appropriately? | □ | □ | □ | x |

Overall appraisal: Include x Exclude □ Seek further info □

Comments (Including reason for exclusion)

______________________________________________________________________________________________________________________________________________________________________________________

JBI Critical Appraisal Checklist for
studies reporting prevalence data

Reviewer Sophie Mathys Date 26.06.2024

Author Jovanovic et al. Year 2015 Record Number_________

|  | Yes | No | Unclear | Not applicable |
| --- | --- | --- | --- | --- |
| 1. Was the sample frame appropriate to address the target population? | x | □ | □ | □ |
| 1. Were study participants sampled in an appropriate way? | □ | □ | x | □ |
| 1. Was the sample size adequate? | x | □ | □ | □ |
| 1. Were the study subjects and the setting described in detail? | x | □ | □ | □ |
| 1. Was the data analysis conducted with sufficient coverage of the identified sample? | □ | □ | x | □ |
| 1. Were valid methods used for the identification of the condition? | x | □ | □ | □ |
| 1. Was the condition measured in a standard, reliable way for all participants? | □ | □ | x | □ |
| 1. Was there appropriate statistical analysis? | □ | □ | x | □ |
| 1. Was the response rate adequate, and if not, was the low response rate managed appropriately? | □ | □ | □ | x |

Overall appraisal: Include x Exclude □ Seek further info □

Comments (Including reason for exclusion)

______________________________________________________________________________________________________________________________________________________________________________________

JBI Critical Appraisal Checklist for
studies reporting prevalence data

Reviewer Sophie Mathys Date 26.06.2024

Author Kaczmarek et al. Year 2023 Record Number_________

|  | Yes | No | Unclear | Not applicable |
| --- | --- | --- | --- | --- |
| 1. Was the sample frame appropriate to address the target population? | x | □ | □ | □ |
| 1. Were study participants sampled in an appropriate way? | x | □ | □ | □ |
| 1. Was the sample size adequate? | x | □ | □ | □ |
| 1. Were the study subjects and the setting described in detail? | x | □ | □ | □ |
| 1. Was the data analysis conducted with sufficient coverage of the identified sample? | □ | □ | x | □ |
| 1. Were valid methods used for the identification of the condition? | x | □ | □ | □ |
| 1. Was the condition measured in a standard, reliable way for all participants? | □ | □ | x | □ |
| 1. Was there appropriate statistical analysis? | □ | □ | x | □ |
| 1. Was the response rate adequate, and if not, was the low response rate managed appropriately? | □ | □ | □ | x |

Overall appraisal: Include x Exclude □ Seek further info □

Comments (Including reason for exclusion)

______________________________________________________________________________________________________________________________________________________________________________________

JBI Critical Appraisal Checklist for
studies reporting prevalence data

Reviewer Sophie Mathys Date 26.06.2024

Author Kalmár et al. Year 2021 Record Number_________

|  | Yes | No | Unclear | Not applicable |
| --- | --- | --- | --- | --- |
| 1. Was the sample frame appropriate to address the target population? | x | □ | □ | □ |
| 1. Were study participants sampled in an appropriate way? | x | □ | □ | □ |
| 1. Was the sample size adequate? | x | □ | □ | □ |
| 1. Were the study subjects and the setting described in detail? | x | □ | □ | □ |
| 1. Was the data analysis conducted with sufficient coverage of the identified sample? | □ | □ | x | □ |
| 1. Were valid methods used for the identification of the condition? | x | □ | □ | □ |
| 1. Was the condition measured in a standard, reliable way for all participants? | □ | □ | x | □ |
| 1. Was there appropriate statistical analysis? | □ | □ | x | □ |
| 1. Was the response rate adequate, and if not, was the low response rate managed appropriately? | □ | □ | □ | x |

Overall appraisal: Include x Exclude □ Seek further info □

Comments (Including reason for exclusion)

______________________________________________________________________________________________________________________________________________________________________________________

JBI Critical Appraisal Checklist for
studies reporting prevalence data

Reviewer Sophie Mathys Date 26.06.2024

Author Koetsveld et al. Year 2016 Record Number_________

|  | Yes | No | Unclear | Not applicable |
| --- | --- | --- | --- | --- |
| 1. Was the sample frame appropriate to address the target population? | x | □ | □ | □ |
| 1. Were study participants sampled in an appropriate way? | □ | □ | x | □ |
| 1. Was the sample size adequate? | □ | □ | x | □ |
| 1. Were the study subjects and the setting described in detail? | x | □ | □ | □ |
| 1. Was the data analysis conducted with sufficient coverage of the identified sample? | □ | □ | x | □ |
| 1. Were valid methods used for the identification of the condition? | x | □ | □ | □ |
| 1. Was the condition measured in a standard, reliable way for all participants? | □ | □ | x | □ |
| 1. Was there appropriate statistical analysis? | □ | □ | x | □ |
| 1. Was the response rate adequate, and if not, was the low response rate managed appropriately? | □ | □ | □ | x |

Overall appraisal: Include x Exclude □ Seek further info □

Comments (Including reason for exclusion)

______________________________________________________________________________________________________________________________________________________________________________________

JBI Critical Appraisal Checklist for
studies reporting prevalence data

Reviewer Sophie Mathys Date 26.06.2024

Author Labbé et al. Year 2022 Record Number_________

|  | Yes | No | Unclear | Not applicable |
| --- | --- | --- | --- | --- |
| 1. Was the sample frame appropriate to address the target population? | x | □ | □ | □ |
| 1. Were study participants sampled in an appropriate way? | x | □ | □ | □ |
| 1. Was the sample size adequate? | x | □ | □ | □ |
| 1. Were the study subjects and the setting described in detail? | x | □ | □ | □ |
| 1. Was the data analysis conducted with sufficient coverage of the identified sample? | □ | □ | x | □ |
| 1. Were valid methods used for the identification of the condition? | x | □ | □ | □ |
| 1. Was the condition measured in a standard, reliable way for all participants? | □ | □ | x | □ |
| 1. Was there appropriate statistical analysis? | □ | □ | x | □ |
| 1. Was the response rate adequate, and if not, was the low response rate managed appropriately? | □ | □ | □ | x |

Overall appraisal: Include x Exclude □ Seek further info □

Comments (Including reason for exclusion)

______________________________________________________________________________________________________________________________________________________________________________________

JBI Critical Appraisal Checklist for
studies reporting prevalence data

Reviewer Sophie Mathys Date 26.06.2024

Author Larsen et al. Year 2014 Record Number_________

|  | Yes | No | Unclear | Not applicable |
| --- | --- | --- | --- | --- |
| 1. Was the sample frame appropriate to address the target population? | x | □ | □ | □ |
| 1. Were study participants sampled in an appropriate way? | x | □ | □ | □ |
| 1. Was the sample size adequate? | x | □ | □ | □ |
| 1. Were the study subjects and the setting described in detail? | x | □ | □ | □ |
| 1. Was the data analysis conducted with sufficient coverage of the identified sample? | □ | □ | x | □ |
| 1. Were valid methods used for the identification of the condition? | x | □ | □ | □ |
| 1. Was the condition measured in a standard, reliable way for all participants? | □ | □ | x | □ |
| 1. Was there appropriate statistical analysis? | □ | □ | x | □ |
| 1. Was the response rate adequate, and if not, was the low response rate managed appropriately? | □ | □ | □ | x |

Overall appraisal: Include x Exclude □ Seek further info □

Comments (Including reason for exclusion)

______________________________________________________________________________________________________________________________________________________________________________________

JBI Critical Appraisal Checklist for
studies reporting prevalence data

Reviewer Sophie Mathys Date 26.06.2024

Author Lindblom et al. Year 2013 Record Number_________

|  | Yes | No | Unclear | Not applicable |
| --- | --- | --- | --- | --- |
| 1. Was the sample frame appropriate to address the target population? | x | □ | □ | □ |
| 1. Were study participants sampled in an appropriate way? | □ | □ | x | □ |
| 1. Was the sample size adequate? | □ | x | □ | □ |
| 1. Were the study subjects and the setting described in detail? | □ | □ | x | □ |
| 1. Was the data analysis conducted with sufficient coverage of the identified sample? | □ | □ | x | □ |
| 1. Were valid methods used for the identification of the condition? | x | □ | □ | □ |
| 1. Was the condition measured in a standard, reliable way for all participants? | □ | □ | x | □ |
| 1. Was there appropriate statistical analysis? | □ | □ | x | □ |
| 1. Was the response rate adequate, and if not, was the low response rate managed appropriately? | □ | □ | □ | x |

Overall appraisal: Include x Exclude □ Seek further info □

Comments (Including reason for exclusion)

______________________________________________________________________________________________________________________________________________________________________________________

JBI Critical Appraisal Checklist for
studies reporting prevalence data

Reviewer Sophie Mathys Date 26.06.2024

Author Łysakowska et al. Year 2019 Record Number_________

|  | Yes | No | Unclear | Not applicable |
| --- | --- | --- | --- | --- |
| 1. Was the sample frame appropriate to address the target population? | x | □ | □ | □ |
| 1. Were study participants sampled in an appropriate way? | x | □ | □ | □ |
| 1. Was the sample size adequate? | x | □ | □ | □ |
| 1. Were the study subjects and the setting described in detail? | x | □ | □ | □ |
| 1. Was the data analysis conducted with sufficient coverage of the identified sample? | □ | □ | x | □ |
| 1. Were valid methods used for the identification of the condition? | x | □ | □ | □ |
| 1. Was the condition measured in a standard, reliable way for all participants? | □ | □ | x | □ |
| 1. Was there appropriate statistical analysis? | □ | □ | x | □ |
| 1. Was the response rate adequate, and if not, was the low response rate managed appropriately? | □ | □ | □ | x |

Overall appraisal: Include x Exclude □ Seek further info □

Comments (Including reason for exclusion)

______________________________________________________________________________________________________________________________________________________________________________________

JBI Critical Appraisal Checklist for
studies reporting prevalence data

Reviewer Sophie Mathys Date 26.06.2024

Author Magyar et al. Year 2021 Record Number_________

|  | Yes | No | Unclear | Not applicable |
| --- | --- | --- | --- | --- |
| 1. Was the sample frame appropriate to address the target population? | x | □ | □ | □ |
| 1. Were study participants sampled in an appropriate way? | x | □ | □ | □ |
| 1. Was the sample size adequate? | x | □ | □ | □ |
| 1. Were the study subjects and the setting described in detail? | □ | □ | x | □ |
| 1. Was the data analysis conducted with sufficient coverage of the identified sample? | □ | □ | x | □ |
| 1. Were valid methods used for the identification of the condition? | x | □ | □ | □ |
| 1. Was the condition measured in a standard, reliable way for all participants? | □ | □ | x | □ |
| 1. Was there appropriate statistical analysis? | □ | □ | x | □ |
| 1. Was the response rate adequate, and if not, was the low response rate managed appropriately? | □ | □ | □ | x |

Overall appraisal: Include x Exclude □ Seek further info □

Comments (Including reason for exclusion)

______________________________________________________________________________________________________________________________________________________________________________________

JBI Critical Appraisal Checklist for
studies reporting prevalence data

Reviewer Sophie Mathys Date 26.06.2024

Author Marvik et al. Year 2021 Record Number_________

|  | Yes | No | Unclear | Not applicable |
| --- | --- | --- | --- | --- |
| 1. Was the sample frame appropriate to address the target population? | x | □ | □ | □ |
| 1. Were study participants sampled in an appropriate way? | □ | □ | x | □ |
| 1. Was the sample size adequate? | x | □ | □ | □ |
| 1. Were the study subjects and the setting described in detail? | □ | □ | x | □ |
| 1. Was the data analysis conducted with sufficient coverage of the identified sample? | □ | □ | x | □ |
| 1. Were valid methods used for the identification of the condition? | x | □ | □ | □ |
| 1. Was the condition measured in a standard, reliable way for all participants? | □ | □ | x | □ |
| 1. Was there appropriate statistical analysis? | □ | □ | x | □ |
| 1. Was the response rate adequate, and if not, was the low response rate managed appropriately? | □ | □ | □ | x |

Overall appraisal: Include x Exclude □ Seek further info □

Comments (Including reason for exclusion)

______________________________________________________________________________________________________________________________________________________________________________________

JBI Critical Appraisal Checklist for
studies reporting prevalence data

Reviewer Sophie Mathys Date 26.06.2024

Author Monsalve et al. Year 2020 Record Number_________

|  | Yes | No | Unclear | Not applicable |
| --- | --- | --- | --- | --- |
| 1. Was the sample frame appropriate to address the target population? | x | □ | □ | □ |
| 1. Were study participants sampled in an appropriate way? | x | □ | □ | □ |
| 1. Was the sample size adequate? | x | □ | □ | □ |
| 1. Were the study subjects and the setting described in detail? | □ | □ | x | □ |
| 1. Was the data analysis conducted with sufficient coverage of the identified sample? | □ | □ | x | □ |
| 1. Were valid methods used for the identification of the condition? | x | □ | □ | □ |
| 1. Was the condition measured in a standard, reliable way for all participants? | □ | □ | x | □ |
| 1. Was there appropriate statistical analysis? | □ | □ | x | □ |
| 1. Was the response rate adequate, and if not, was the low response rate managed appropriately? | □ | □ | □ | x |

Overall appraisal: Include x Exclude □ Seek further info □

Comments (Including reason for exclusion)

______________________________________________________________________________________________________________________________________________________________________________________

JBI Critical Appraisal Checklist for
studies reporting prevalence data

Reviewer Sophie Mathys Date 26.06.2024

Author Müller et al. Year 2016 Record Number_________

|  | Yes | No | Unclear | Not applicable |
| --- | --- | --- | --- | --- |
| 1. Was the sample frame appropriate to address the target population? | x | □ | □ | □ |
| 1. Were study participants sampled in an appropriate way? | □ | □ | x | □ |
| 1. Was the sample size adequate? | x | □ | □ | □ |
| 1. Were the study subjects and the setting described in detail? | □ | □ | x | □ |
| 1. Was the data analysis conducted with sufficient coverage of the identified sample? | □ | □ | x | □ |
| 1. Were valid methods used for the identification of the condition? | x | □ | □ | □ |
| 1. Was the condition measured in a standard, reliable way for all participants? | □ | □ | x | □ |
| 1. Was there appropriate statistical analysis? | □ | □ | x | □ |
| 1. Was the response rate adequate, and if not, was the low response rate managed appropriately? | □ | □ | □ | x |

Overall appraisal: Include x Exclude □ Seek further info □

Comments (Including reason for exclusion)

______________________________________________________________________________________________________________________________________________________________________________________

JBI Critical Appraisal Checklist for
studies reporting prevalence data

Reviewer Sophie Mathys Date 26.06.2024

Author Munro et al. Year 2015 Record Number_________

|  | Yes | No | Unclear | Not applicable |
| --- | --- | --- | --- | --- |
| 1. Was the sample frame appropriate to address the target population? | x | □ | □ | □ |
| 1. Were study participants sampled in an appropriate way? | □ | □ | x | □ |
| 1. Was the sample size adequate? | x | □ | □ | □ |
| 1. Were the study subjects and the setting described in detail? | □ | □ | x | □ |
| 1. Was the data analysis conducted with sufficient coverage of the identified sample? | □ | □ | x | □ |
| 1. Were valid methods used for the identification of the condition? | x | □ | □ | □ |
| 1. Was the condition measured in a standard, reliable way for all participants? | □ | □ | x | □ |
| 1. Was there appropriate statistical analysis? | □ | □ | x | □ |
| 1. Was the response rate adequate, and if not, was the low response rate managed appropriately? | □ | □ | □ | x |

Overall appraisal: Include x Exclude □ Seek further info □

Comments (Including reason for exclusion)

______________________________________________________________________________________________________________________________________________________________________________________

JBI Critical Appraisal Checklist for
studies reporting prevalence data

Reviewer Sophie Mathys Date 26.06.2024

Author Mygland et al. Year 2006 Record Number_________

|  | Yes | No | Unclear | Not applicable |
| --- | --- | --- | --- | --- |
| 1. Was the sample frame appropriate to address the target population? | x | □ | □ | □ |
| 1. Were study participants sampled in an appropriate way? | □ | □ | x | □ |
| 1. Was the sample size adequate? | x | □ | □ | □ |
| 1. Were the study subjects and the setting described in detail? | □ | □ | x | □ |
| 1. Was the data analysis conducted with sufficient coverage of the identified sample? | □ | □ | x | □ |
| 1. Were valid methods used for the identification of the condition? | x | □ | □ | □ |
| 1. Was the condition measured in a standard, reliable way for all participants? | □ | □ | x | □ |
| 1. Was there appropriate statistical analysis? | □ | □ | x | □ |
| 1. Was the response rate adequate, and if not, was the low response rate managed appropriately? | □ | □ | □ | x |

Overall appraisal: Include x Exclude □ Seek further info □

Comments (Including reason for exclusion)

______________________________________________________________________________________________________________________________________________________________________________________

JBI Critical Appraisal Checklist for
studies reporting prevalence data

Reviewer Sophie Mathys Date 26.06.2024

Author Ocias et al. Year 2018 Record Number_________

|  | Yes | No | Unclear | Not applicable |
| --- | --- | --- | --- | --- |
| 1. Was the sample frame appropriate to address the target population? | x | □ | □ | □ |
| 1. Were study participants sampled in an appropriate way? | □ | □ | x | □ |
| 1. Was the sample size adequate? | □ | □ | x | □ |
| 1. Were the study subjects and the setting described in detail? | □ | □ | x | □ |
| 1. Was the data analysis conducted with sufficient coverage of the identified sample? | □ | □ | x | □ |
| 1. Were valid methods used for the identification of the condition? | x | □ | □ | □ |
| 1. Was the condition measured in a standard, reliable way for all participants? | □ | □ | x | □ |
| 1. Was there appropriate statistical analysis? | □ | □ | x | □ |
| 1. Was the response rate adequate, and if not, was the low response rate managed appropriately? | □ | □ | □ | x |

Overall appraisal: Include x Exclude □ Seek further info □

Comments (Including reason for exclusion)

______________________________________________________________________________________________________________________________________________________________________________________

JBI Critical Appraisal Checklist for
studies reporting prevalence data

Reviewer Sophie Mathys Date 26.06.2024

Author Pawełczyk et al. Year 2019 Record Number_________

|  | Yes | No | Unclear | Not applicable |
| --- | --- | --- | --- | --- |
| 1. Was the sample frame appropriate to address the target population? | x | □ | □ | □ |
| 1. Were study participants sampled in an appropriate way? | □ | □ | x | □ |
| 1. Was the sample size adequate? | □ | □ | x | □ |
| 1. Were the study subjects and the setting described in detail? | □ | □ | x | □ |
| 1. Was the data analysis conducted with sufficient coverage of the identified sample? | □ | □ | x | □ |
| 1. Were valid methods used for the identification of the condition? | x | □ | □ | □ |
| 1. Was the condition measured in a standard, reliable way for all participants? | □ | □ | x | □ |
| 1. Was there appropriate statistical analysis? | □ | □ | x | □ |
| 1. Was the response rate adequate, and if not, was the low response rate managed appropriately? | □ | □ | □ | x |

Overall appraisal: Include x Exclude □ Seek further info □

Comments (Including reason for exclusion)

______________________________________________________________________________________________________________________________________________________________________________________

JBI Critical Appraisal Checklist for
studies reporting prevalence data

Reviewer Sophie Mathys Date 26.06.2024

Author Pomelova et al. Year 2015 Record Number_________

|  | Yes | No | Unclear | Not applicable |
| --- | --- | --- | --- | --- |
| 1. Was the sample frame appropriate to address the target population? | x | □ | □ | □ |
| 1. Were study participants sampled in an appropriate way? | □ | □ | x | □ |
| 1. Was the sample size adequate? | x | □ | □ | □ |
| 1. Were the study subjects and the setting described in detail? | x | □ | □ | □ |
| 1. Was the data analysis conducted with sufficient coverage of the identified sample? | □ | □ | x | □ |
| 1. Were valid methods used for the identification of the condition? | x | □ | □ | □ |
| 1. Was the condition measured in a standard, reliable way for all participants? | □ | □ | x | □ |
| 1. Was there appropriate statistical analysis? | □ | □ | x | □ |
| 1. Was the response rate adequate, and if not, was the low response rate managed appropriately? | □ | □ | □ | x |

Overall appraisal: Include x Exclude □ Seek further info □

Comments (Including reason for exclusion)

______________________________________________________________________________________________________________________________________________________________________________________

JBI Critical Appraisal Checklist for
studies reporting prevalence data

Reviewer Sophie Mathys Date 26.06.2024

Author Santos et al. Year 2006 Record Number_________

|  | Yes | No | Unclear | Not applicable |
| --- | --- | --- | --- | --- |
| 1. Was the sample frame appropriate to address the target population? | x | □ | □ | □ |
| 1. Were study participants sampled in an appropriate way? | □ | □ | x | □ |
| 1. Was the sample size adequate? | □ | x | □ | □ |
| 1. Were the study subjects and the setting described in detail? | □ | □ | x | □ |
| 1. Was the data analysis conducted with sufficient coverage of the identified sample? | □ | □ | x | □ |
| 1. Were valid methods used for the identification of the condition? | x | □ | □ | □ |
| 1. Was the condition measured in a standard, reliable way for all participants? | □ | □ | x | □ |
| 1. Was there appropriate statistical analysis? | □ | □ | x | □ |
| 1. Was the response rate adequate, and if not, was the low response rate managed appropriately? | □ | □ | □ | x |

Overall appraisal: Include x Exclude □ Seek further info □

Comments (Including reason for exclusion)

______________________________________________________________________________________________________________________________________________________________________________________

JBI Critical Appraisal Checklist for
studies reporting prevalence data

Reviewer Sophie Mathys Date 26.06.2024

Author Sonnleitner et al. Year 2013 Record Number_________

|  | Yes | No | Unclear | Not applicable |
| --- | --- | --- | --- | --- |
| 1. Was the sample frame appropriate to address the target population? | x | □ | □ | □ |
| 1. Were study participants sampled in an appropriate way? | x | □ | □ | □ |
| 1. Was the sample size adequate? | x | □ | □ | □ |
| 1. Were the study subjects and the setting described in detail? | x | □ | □ | □ |
| 1. Was the data analysis conducted with sufficient coverage of the identified sample? | □ | □ | x | □ |
| 1. Were valid methods used for the identification of the condition? | x | □ | □ | □ |
| 1. Was the condition measured in a standard, reliable way for all participants? | □ | □ | x | □ |
| 1. Was there appropriate statistical analysis? | □ | □ | x | □ |
| 1. Was the response rate adequate, and if not, was the low response rate managed appropriately? | □ | □ | □ | x |

Overall appraisal: Include x Exclude □ Seek further info □

Comments (Including reason for exclusion)

______________________________________________________________________________________________________________________________________________________________________________________

JBI Critical Appraisal Checklist for
studies reporting prevalence data

Reviewer Sophie Mathys Date 26.06.2024

Author Sonnleitner et al. Year 2015 Record Number_________

|  | Yes | No | Unclear | Not applicable |
| --- | --- | --- | --- | --- |
| 1. Was the sample frame appropriate to address the target population? | x | □ | □ | □ |
| 1. Were study participants sampled in an appropriate way? | x | □ | □ | □ |
| 1. Was the sample size adequate? | x | □ | □ | □ |
| 1. Were the study subjects and the setting described in detail? | □ | □ | x | □ |
| 1. Was the data analysis conducted with sufficient coverage of the identified sample? | □ | □ | x | □ |
| 1. Were valid methods used for the identification of the condition? | x | □ | □ | □ |
| 1. Was the condition measured in a standard, reliable way for all participants? | □ | □ | x | □ |
| 1. Was there appropriate statistical analysis? | □ | □ | x | □ |
| 1. Was the response rate adequate, and if not, was the low response rate managed appropriately? | □ | □ | □ | x |

Overall appraisal: Include x Exclude □ Seek further info □

Comments (Including reason for exclusion)

______________________________________________________________________________________________________________________________________________________________________________________

JBI Critical Appraisal Checklist for
studies reporting prevalence data

Reviewer Sophie Mathys Date 26.06.2024

Author Sonnleitner et al. Year 2014 Record Number_________

|  | Yes | No | Unclear | Not applicable |
| --- | --- | --- | --- | --- |
| 1. Was the sample frame appropriate to address the target population? | x | □ | □ | □ |
| 1. Were study participants sampled in an appropriate way? | x | □ | □ | □ |
| 1. Was the sample size adequate? | x | □ | □ | □ |
| 1. Were the study subjects and the setting described in detail? | x | □ | □ | □ |
| 1. Was the data analysis conducted with sufficient coverage of the identified sample? | □ | □ | x | □ |
| 1. Were valid methods used for the identification of the condition? | x | □ | □ | □ |
| 1. Was the condition measured in a standard, reliable way for all participants? | □ | □ | x | □ |
| 1. Was there appropriate statistical analysis? | □ | □ | x | □ |
| 1. Was the response rate adequate, and if not, was the low response rate managed appropriately? | □ | □ | □ | x |

Overall appraisal: Include x Exclude □ Seek further info □

Comments (Including reason for exclusion)

______________________________________________________________________________________________________________________________________________________________________________________

JBI Critical Appraisal Checklist for
studies reporting prevalence data

Reviewer Sophie Mathys Date 26.06.2024

Author Stańczak et al. Year 2016 Record Number_________

|  | Yes | No | Unclear | Not applicable |
| --- | --- | --- | --- | --- |
| 1. Was the sample frame appropriate to address the target population? | x | □ | □ | □ |
| 1. Were study participants sampled in an appropriate way? | □ | □ | x | □ |
| 1. Was the sample size adequate? | □ | x | □ | □ |
| 1. Were the study subjects and the setting described in detail? | x | □ | □ | □ |
| 1. Was the data analysis conducted with sufficient coverage of the identified sample? | □ | □ | x | □ |
| 1. Were valid methods used for the identification of the condition? | x | □ | □ | □ |
| 1. Was the condition measured in a standard, reliable way for all participants? | □ | □ | x | □ |
| 1. Was there appropriate statistical analysis? | □ | □ | x | □ |
| 1. Was the response rate adequate, and if not, was the low response rate managed appropriately? | □ | □ | □ | x |

Overall appraisal: Include x Exclude □ Seek further info □

Comments (Including reason for exclusion)

______________________________________________________________________________________________________________________________________________________________________________________

JBI Critical Appraisal Checklist for
studies reporting prevalence data

Reviewer Sophie Mathys Date 26.06.2024

Author Tomasiewicz et al. Year 2004 Record Number_________

|  | Yes | No | Unclear | Not applicable |
| --- | --- | --- | --- | --- |
| 1. Was the sample frame appropriate to address the target population? | x | □ | □ | □ |
| 1. Were study participants sampled in an appropriate way? | □ | □ | x | □ |
| 1. Was the sample size adequate? | □ | x | □ | □ |
| 1. Were the study subjects and the setting described in detail? | □ | □ | x | □ |
| 1. Was the data analysis conducted with sufficient coverage of the identified sample? | □ | □ | x | □ |
| 1. Were valid methods used for the identification of the condition? | x | □ | □ | □ |
| 1. Was the condition measured in a standard, reliable way for all participants? | □ | □ | x | □ |
| 1. Was there appropriate statistical analysis? | □ | □ | x | □ |
| 1. Was the response rate adequate, and if not, was the low response rate managed appropriately? | □ | □ | □ | x |

Overall appraisal: Include x Exclude □ Seek further info □

Comments (Including reason for exclusion)

______________________________________________________________________________________________________________________________________________________________________________________

JBI Critical Appraisal Checklist for
studies reporting prevalence data

Reviewer Sophie Mathys Date 26.06.2024

Author Walder et al. Year 2003 Record Number_________

|  | Yes | No | Unclear | Not applicable |
| --- | --- | --- | --- | --- |
| 1. Was the sample frame appropriate to address the target population? | x | □ | □ | □ |
| 1. Were study participants sampled in an appropriate way? | □ | □ | x | □ |
| 1. Was the sample size adequate? | x | □ | □ | □ |
| 1. Were the study subjects and the setting described in detail? | □ | □ | x | □ |
| 1. Was the data analysis conducted with sufficient coverage of the identified sample? | □ | □ | x | □ |
| 1. Were valid methods used for the identification of the condition? | x | □ | □ | □ |
| 1. Was the condition measured in a standard, reliable way for all participants? | □ | □ | x | □ |
| 1. Was there appropriate statistical analysis? | □ | □ | x | □ |
| 1. Was the response rate adequate, and if not, was the low response rate managed appropriately? | □ | □ | □ | x |

Overall appraisal: Include x Exclude □ Seek further info □

Comments (Including reason for exclusion)

______________________________________________________________________________________________________________________________________________________________________________________

JBI Critical Appraisal Checklist for
studies reporting prevalence data

Reviewer Sophie Mathys Date 26.06.2024

Author Zákutná et al. Year 2015 Record Number_________

|  | Yes | No | Unclear | Not applicable |
| --- | --- | --- | --- | --- |
| 1. Was the sample frame appropriate to address the target population? | x | □ | □ | □ |
| 1. Were study participants sampled in an appropriate way? | □ | □ | x | □ |
| 1. Was the sample size adequate? | x | □ | □ | □ |
| 1. Were the study subjects and the setting described in detail? | □ | □ | x | □ |
| 1. Was the data analysis conducted with sufficient coverage of the identified sample? | □ | □ | x | □ |
| 1. Were valid methods used for the identification of the condition? | x | □ | □ | □ |
| 1. Was the condition measured in a standard, reliable way for all participants? | □ | □ | x | □ |
| 1. Was there appropriate statistical analysis? | □ | □ | x | □ |
| 1. Was the response rate adequate, and if not, was the low response rate managed appropriately? | □ | □ | □ | x |

Overall appraisal: Include x Exclude □ Seek further info □

Comments (Including reason for exclusion)

______________________________________________________________________________________________________________________________________________________________________________________
